# Treatment escalation and de-escalation decisions in Crohn’s disease: Delphi consensus recommendations from Japan, 2021

**DOI:** 10.1007/s00535-023-01958-z

**Published:** 2023-02-11

**Authors:** Hiroshi Nakase, Motohiro Esaki, Fumihito Hirai, Taku Kobayashi, Katsuyoshi Matsuoka, Minoru Matsuura, Makoto Naganuma, Masayuki Saruta, Kiichiro Tsuchiya, Motoi Uchino, Kenji Watanabe, Tadakazu Hisamatsu, the TRADE consensus group, Akira Andoh, Shigeki Bamba, Motohiro Esaki, Mikihiro Fujiya, Kitaro Futami, Keisuke Hata, Fumihito Hirai, Sakiko Hiraoka, Tadakazu Hisamatsu, Ryota Hokari, Shunji Ishihara, Soichiro Ishihara, Michio Itabashi, Yoichi Kakuta, Jun Kato, Shingo Kato, Takehiko Katsurada, Kazuya Kitamura, Kiyonori Kobayashi, Taku Kobayashi, Kazutaka Koganei, Atsuo Maemoto, Toshiyuki Matsui, Takayuki Matsumoto, Katsuyoshi Matsuoka, Minoru Matsuura, Satoshi Motoya, Masakazu Nagahori, Makoto Naganuma, Yuji Naito, Shiro Nakamura, Hiroshi Nakase, Haruhiko Ogata, Kazuichi Okazaki, Hirotake Sakuraba, Masayuki Saruta, Shinichiro Shinzaki, Ken Sugimoto, Akira Sugita, Yasuo Suzuki, Kenichi Takahashi, Tomohisa Takagi, Kento Takenaka, Ken Takeuchi, Kiichiro Tsuchiya, Tomoyuki Tsujikawa, Motoi Uchino, Fumiaki Ueno, Kenji Watanabe, Mamoru Watanabe, Takayuki Yamamoto, Kaoru Yokoyama, Atsushi Yoshida, Naoki Yoshimura

**Affiliations:** 1 Department of Gastroenterology and Hepatology, Sapporo Medical University School of Medicine, South-1, West-16, Chuo-Ku, Sapporo, Hokkaido 060-8543 Japan; 2 Division of Gastroenterology, Department of Internal Medicine, Faculty of Medicine, Saga University, Saga, Japan; 3 Department of Gastroenterology and Medicine, Faculty of Medicine, Fukuoka University, Fukuoka, Japan; 4 Center for Advanced IBD Research and Treatment, Kitasato University, Kitasato Institute Hospital, Tokyo, Japan; 5 Division of Gastroenterology and Hepatology, Department of Internal Medicine, Toho University Sakura Medical Center, Sakura, Chiba Japan; 6 Department of Gastroenterology and Hepatology, Kyorin University School of Medicine, 6-20-2 Shinkawa, Mitaka-Shi, Tokyo, 181-8611 Japan; 7 Division of Gastroenterology and Hepatology, The Third Department of Internal Medicine, Kansai Medical University, Osaka, Japan; 8 Division of Gastroenterology and Hepatology, Department of Internal Medicine, The Jikei University School of Medicine, Tokyo, Japan; 9 Department of Gastroenterology, Faculty of Medicine, University of Tsukuba, Tsukuba, Japan; 10 Division of Inflammatory Bowel Disease, Department of Gastroenterological Surgery, Hyogo Medical University, Nishinomiya, Hyogo Japan; 11 Division of Gastroenterology and Hepatology, Department of Internal Medicine, Hyogo Medical University, Nishinomiya, Hyogo Japan

**Keywords:** Consensus, Crohn’s disease, Luminal disease, Treatment escalation/de-escalation

## Abstract

**Background:**

We aimed to develop criteria for treatment intensification in patients with (1) luminal Crohn’s disease (CD), (2) CD with perianal disease and/or fistula, (3) CD with small bowel stenosis, (4) in the postoperative setting, and (5) for discontinuing or reducing the dose of treatment in patients with CD.

**Methods:**

PubMed and Embase were searched for studies published since 1998 which may be relevant to the five defined topics. Results were assessed for relevant studies, with preference given to data from randomized, controlled studies. For each question, a core panel of 12 gastroenterologists defined the treatment target and developed statements, based on the literature, current guidelines, and relevant additional studies. The evidence supporting each statement was graded using the Oxford Centre for Evidence-Based Medicine: Levels of Evidence (March 2009). A modified Delphi process was used to refine statements and gain agreement from 54 Japanese specialists at in-person and online meetings conducted between October 2020 and April 2021.

**Results:**

Seventeen statements were developed for treatment intensification in luminal CD (targeting endoscopic remission), six statements for treatment intensification in perianal/fistulizing CD (targeting healing of perianal lesions and complete closure of the fistula), six statements for treatment intensification in CD with small bowel stenosis (targeting resolution of obstructive symptoms), seven statements for treatment intensification after surgery (targeting endoscopic remission), and five statements for discontinuing or reducing the dose of treatment in patients with CD.

**Conclusions:**

These statements provide guidance on how and when to intensify or de-intensify treatment for a broad spectrum of patients with CD.

**Supplementary Information:**

The online version contains supplementary material available at 10.1007/s00535-023-01958-z.

## Introduction

Various guidelines exist on the management of Crohn’s disease (CD), with most advocating a ‘treat-to-target’ (T2T) approach to medical management [1–9]. The International Organization for the Study of Inflammatory Bowel Diseases (IOIBD) initiated the Selecting Therapeutic Targets in Inflammatory Bowel Disease (STRIDE) project to define these targets [2, 10]. The major features of the recent STRIDE-II recommendations are the definition of stepwise targets (specifically short-, intermediate-, and long-term treatment targets) in a T2T approach aimed at improving the long-term quality of life of patients with inflammatory bowel disease; and the use of biomarkers to confirm intermediate targets during T2T.

STRIDE-II offers a practical strategy with optimized objective monitoring by biomarkers and imaging modalities including endoscopy. However, whether we can apply it to all phenotypes of CD, including complicated disease, is an issue for further study.

While the STRIDE targets for CD are important for defining the desired outcome of treatment, they do not provide specific information about when to escalate therapy in the pursuit of that outcome. Such information is important for physicians in clinical practice who must make clinical decisions based not only on treatment goals, but on the clinical, physical, psychosocial, economic, and emotional status of each patient they manage. The TReatment escAlation and DE-escalation decisions in Crohn’s disease (TRADE) group was convened to help answer questions about the criteria for treatment intensification in patients with luminal CD, CD with perianal/fistulizing disease or small bowel stenosis, and in the postoperative setting, and the criteria for discontinuing or reducing the dose of treatment (i.e., de-escalation) in patients with CD. The TRADE group used a modified Delphi consensus process to development statements relating to each question.

## Methods

A core group of 12 Japanese gastroenterology specialists (comprising two co-chairs and ten members) was convened to oversee the development of consensus recommendations to answer the following five questions:

1. What are the criteria for treatment intensification in patients with luminal CD?
2. What are the criteria for treatment intensification in patients with CD with perianal or fistulizing disease?
3. What are the criteria for treatment intensification in patients with CD with small bowel stenosis?
4. What considerations are essential for treatment intensification in patients with CD in the postoperative setting?
5. What are the considerations for de-escalation (i.e., discontinuing or reducing the dose of treatment) in patients with CD?

In addition to the core group, an additional 42 gastroenterologists (Supplementary Table 1) were selected for the Delphi panel based on their expertise and specialist knowledge. These 54 individuals comprised the TRADE group. All members of the TRADE group specialized in the management of inflammatory bowel disease (IBD) [as surgeons, endoscope specialists, and physicians], worked at tertiary hospitals, medical centers or clinics in Japan, and were familiar with Japanese clinical practice guidelines relating to IBD.

For each question, a literature search of Embase and Medline was conducted in December 2019. The first four searches used key words related to CD and treatment intensification or escalation, and the searches for questions 2, 3 and 4 included key words related to perianal/anal involvement, stenosis, and surgery, respectively. The fifth search used key words related to CD and treatment de-escalation or discontinuation. All searches were limited to studies in human subjects published in English. The search period was the end of 1998 to December 2019 for searches 2, 3 and 4, and the end of 2014 to December 2019 for searches 1 and 5, due to the large volume of data published on these latter topics. Complete details of the literature search and results are shown in the Supplementary Table 2.

The search results were assessed by the core group for relevant studies based on the title and abstract, with preference given to data from randomized, controlled studies. For each question, the treatment target was defined and statements were developed, based on the literature results, current guidelines, and relevant additional studies known to the authors. The evidence supporting each statement was graded using the Oxford Centre for Evidence-Based Medicine: Levels of Evidence (March 2009) [11].

Four online meetings of the TRADE group, including the 12 core members, were held between 06 June and 07 August, 2020 to develop the statements. Each meeting related to one question (question 1) or two questions, with questions 2 and 4 covered in two separate meetings. At each meeting, two individuals were elected as section leaders at each meeting (with the exception of the meeting for question 1, for which there were four leaders) who were responsible for drafting the statements relevant to each question. The corresponding authors of this paper attended all meetings. Consensus for each statement was subsequently assessed at a meeting on 03 October 2020. Fifty-one of the 54 gastroenterologists in the TRADE group (eleven individuals from the core group and 40 member gastroenterologists) attended the meeting in October 2020 and acted as a survey panel. This meeting consisted of two parts: during the first part, panel members discussed each statement in detail and made amendments where necessary; in the second part, the revised statements were presented for voting. In the latter part of the meeting, the panel used a web-based digital survey system to express their agreement or disagreement with each statement, using a 9-point scale (1, least agree to 9, most agree, with scores 7, 8, 9 defined as ‘agree’ for the purposes of consensus). The results of the voting were used to determine the level of consensus for each statement. Two rounds of voting were held, with consensus defined by a specified percentage of respondents scoring the statements as 7, 8, or 9. In the first round, a consensus of ≥ 75% led to inclusion of the statement and < 75% led to the second round of voting, which used the same score inclusion policy. The voting and consensus rates are shown in Supplementary Table 3. A consensus was reached for all statements. Final statements and the rationale for each statement were circulated to 54 members for final approval on 30 April 2021.

Consensus statements arising from each question are presented below followed by a discussion supporting these recommendations.

## Consensus recommendations

### Question 1. What are the criteria for treatment intensification in patients with luminal CD?

Target: endoscopic remission.

**Table:** 

Statement 1.1: Initial identification of primary non-response or loss of response after initial improvement is based on signs and symptoms, biomarkers and global assessment of the patient’s condition, including extraintestinal manifestations, by the treating physician (Evidence level 1a)	Voting agreement rate 41/46 (89.1%)

Non-response to medical therapy, whether primary or after an initial remission/improvement, is the most commonly cited reason for treatment escalation in the literature [6, 12–47]. Real-world evidence also suggests that dose escalation is associated with shorter disease duration and corticosteroid use and occurs at a lower frequency in clinical practice than in trial settings [48]. Suboptimal response is usually based on signs and symptoms of CD (such as weight loss, diarrhea, abdominal pain), extraintestinal manifestations (e.g., iritis or arthritis), use of symptomatic treatments (e.g., for diarrhea or pain), blood test parameters (e.g., systemic markers of inflammation such as C-reactive protein [CRP]), and patient general well-being. While some research has been undertaken to develop a clinical risk score that will help to identify patients who would benefit from treatment escalation [49], there is currently no accepted and validated scoring system for this purpose that is recommended in clinical guidelines.

**Table:** 

Statement 1.2: The presence of extraintestinal manifestations is often a sign of ongoing intestinal inflammation and may indicate a need for more intensive therapy (Evidence level 2b)	Voting agreement rate 42/44 (95.5%)

Up to 50% of patients with CD develop extraintestinal manifestations of the disease; the most common types being arthropathy, dermatological conditions (e.g., erythema nodosum, pyoderma gangrenosum, Sweet’s syndrome), oral aphthous ulcers, ocular conditions (e.g., episcleritis or uveitis) and hepatobiliary conditions (most commonly primary sclerosing cholangitis) [50]. These conditions may indicate intestinal inflammation, but can also occur independently (Supplementary Table 4) [50].

Given the potential relationship between extraintestinal manifestations and bowel inflammation, the presence of extraintestinal manifestations may prompt more intensive anti-inflammatory therapy [51]. Patients with extraintestinal manifestations may also benefit from referral to another specialist (e.g., dermatologist, ophthalmologist, rheumatologist) and local and/or symptomatic therapies [51].

**Table:** 

Statement 1.3: The Crohn’s Disease Activity Index (CDAI) or Harvey–Bradshaw Index (HBI) can be used to measure clinical disease activity, with both measures being equally effective. Because of its simplicity, the HBI is recommended as the instrument of choice for assessing clinical disease activity in routine clinical practice; an HBI score of > 4 is indicative of active disease (Evidence level 1b, 5)	Voting agreement rate 42/44 (95.5%)

Clinical studies in patients with CD often use the Crohn’s Disease Activity Index (CDAI in adults or Pediatric CDAI [PCDAI] in children) to assess signs and symptoms (Supplementary Table 5) [52]. A CDAI score of < 150 is used to define remission [5, 53]. A recent literature survey in Japan found that the CDAI was the most commonly used index in clinical trials [54]. However, the threshold for treatment escalation based on CDAI score differs between studies. Several randomized controlled trials published since 2014 used the CDAI as part of the criteria to define escalation [14, 55–58]. In their 2008 study comparing a top-down vs a step-up approach to CD treatment, D’Haens and colleagues used an increase in CDAI score of ≥ 50 points to escalate treatment [57]. The GAIN study used a CDAI score of 220–450 in patients already taking infliximab to identify those who may benefit from a change in anti-TNFα therapy [58]. In the CALM study, the CDAI score used to define moderate to severe CD in the patient inclusion criteria was 220–450 in patients not receiving prednisone at baseline, 200–450 in patients receiving prednisone at a dose of ≤ 20 mg for ≥ 7 days before baseline, and > 150 to 450 in patients receiving prednisone at a dose of > 20 mg for ≥ 7 days before baseline [14]. The TAILORIX study used a hierarchical approach to treatment escalation criteria; the first step in this hierarchy was a CDAI score > 220 and the second step used a CDAI score of 150–220 for two consecutive weeks [55]. The only recent randomized trial in children used a PCDAI score of ≥ 10 to define loss of response [12]. In all of these studies, CDAI criteria alone were insufficient to consider treatment escalation (see further discussion below) [12, 14, 55, 56].

The two predominant symptoms measured in the CDAI (stool frequency and abdominal pain) are individually only weakly correlated with endoscopic findings, but the correlation tends to be a little stronger for stool frequency (*r* = 0.36) than for abdominal pain (*r* = 0.22) [59]. Presumably, this is because pain is complex and has many potential causes including some unrelated to active inflammation (e.g., disrupted motility, fibrosis, adhesions, strictures) [59].

While the CDAI is widely used in a clinical research context, it may not be practical for use in clinical practice, because it is complicated to calculate, varies between patients (especially in relation to items such as abdominal pain) and relies on patients completing symptom diaries, uses a poorly defined standard for body weight, does not accurately assess the severity of fistulas and stenosis, and is weighted heavily toward diarrheal symptoms [1, 54]. The simpler Harvey–Bradshaw index (HBI) may be a more suitable tool for the assessment of disease activity in clinical practice (Supplementary Table 6), but this is also weighted by diarrhea meaning that CD patients with an increased stool frequency are likely to show a score disproportionate to disease activity [1, 54]. Studies using the HBI generally define lack of response as a score of ≥ 5 [28, 42], although some have used a score of > 4 [60, 61]. HBI scores are highly correlated with CDAI scores (correlation coefficient of 0.8–0.93) [61, 62]; an HBI score > 4 is equivalent to a CDAI score of > 150 [61].

**Table:** 

Statement 1.4: While disease signs and symptoms are often the first indicator that treatment is suboptimal, treatment escalation should not be undertaken without confirming the presence of ongoing intestinal inflammation (Evidence level 3, 5)	Voting agreement rate 44/44 (100%)

In clinical practice, physicians generally do not use a formal tool to assess disease activity, but rather assess their patient’s condition based on physical examination, patient report, and blood test results. This is a valid approach, but decisions on treatment escalation should not be solely based on signs of disease activity, without confirming the presence of ongoing intestinal inflammation. Neither clinical signs of disease activity nor patient reports of worsening symptoms correlate strongly with ongoing inflammation in CD [1, 63, 64]. Some CD patients have concomitant functional bowel conditions that can worsen symptoms in the absence of ongoing inflammation [65]. Conversely, some patients in clinical remission may have mucosal inflammation [65], as demonstrated in the ACCENT 1 study [66]. The STRIDE II guidelines for treatment outcomes highlight the importance of endoscopic healing as an intermediate-to-long-term treatment goal, alongside the need to resolve/minimize symptoms in the short term [10]. A recent retrospective study of CD patients found that, despite a moderate level of agreement with endoscopic healing, histologic healing was more closely associated with decreased risk of clinical relapse, medication escalation, or corticosteroid use [67]. As noted in a recent review, histological remission is increasingly becoming a novel target within the concept of disease clearance comprising clinical, endoscopic and microscopic remission [68]. However, the extent to which this more rigorous treatment goal can be adopted in daily clinical practice requires further examination.

**Table:** 

Statement 1.5: Patients with worsening signs or symptoms of CD during treatment should undergo assessment for intestinal inflammation, using biomarkers, small bowel radiography, endoscopy or cross-sectional imaging (computed tomography [CT] or magnetic resonance imaging [MRI]) (Evidence level 5)	Voting agreement rate 45/45 (100%)
Statement 1.6: Endoscopy is the established standard for the assessment of intestinal inflammation (Evidence level 5)	46/47 (97.9%)
Statement 1.7: It is important to visualize the small bowel mucosa, using balloon-assisted endoscopy or capsule endoscopy, when active small bowel disease is suspected (Evidence level 2a)	47/47 (100%)
Statement 1.8: When endoscopy or capsule endoscopy is not feasible or practical, noninvasive imaging using magnetic resonance enterography (MRE), CT enterography (CTE) or ultrasound is the preferred modality (Evidence level 5)	47/47 (100%)
Statement 1.9: CT use should be minimized in patients with CD to limit lifetime exposure to radiation (Evidence level 1a/1b)	47/47 (100%)

For objective assessment of intestinal inflammation, UK guidelines and STRIDE I/II recommendations recommend endoscopy as the preferred modality [1, 2, 10]. In Japan, the most common type of CD is ileocolonic [69, 70], so ileocolonoscopy is the most widely used modality. However, many Japanese patients (between 16% and 36%) do not have colonic involvement [69, 70]. Ileocolonoscopy does not reach portions of the bowel proximal to the terminal ileum. Therefore, in patients with known small bowel disease or upper gastrointestinal symptoms (e.g., anemia, weight loss, cramping after meals, nausea/vomiting, upper abdominal pain), other modalities that visualize the small bowel may be needed. For these patients, physicians can use small bowel radiography, capsule endoscopy or balloon-assisted endoscopy to assess the small bowel [1, 71–74].

Capsule endoscopy is a valid, noninvasive alternative to conventional endoscopy, and has a sensitivity of 83% and specificity of 53% for the diagnosis of active CD in the small bowel [75]. However, there are currently no validated criteria for the interpretation of capsule endoscopy and there is a small risk of retention, particularly in patients with strictures [1]. A few studies identified in our search used capsule endoscopy to determine treatment decisions [76–80]. In one prospective observational study, patients aged ≥ 10 years with CD and a Lewis score of > 135 on capsule endoscopy could have their treatment escalated, irrespective of symptoms [76]. Of 40 patients, 29 (72.5%) patients underwent treatment escalation and experienced an improvement in CDAI and Lewis score. While these data are promising, the study was small, not randomized, and there was no control group to assess outcomes in patients with a high Lewis score who did not undergo treatment escalation. Another observational study found that panenteric capsule endoscopy resulted in treatment intensification in 38.7% of patients overall but especially among those with an established diagnosis whose disease was active (64.6% of patients) [80]. A Japanese study analyzed the correlations between several biomarkers and the Capsule Endoscopy Crohn's Disease Activity Index (CECDAI) or Lewis score [77]. This found that capsule endoscopy could identify intestinal abnormalities in most CD patients in clinical remission with detection rates using CECDAI (81.0%) and the Lewis score (85.7%) being highly correlated. Finally, a retrospective single-center study from Japan found that patients with a Lewis score > 270 (and Prognostic Nutritional Index < 45) were at increased risk of exacerbation, prompting the need for treatment escalation at these thresholds [78]. Among pediatric patients, a new score (Capsule endoscopy–Crohn’s disease index, CE–CD) reliably and simply predicted the need for treatment escalation in this specific population [79].

However, the extent of intestinal inflammation can also be assessed using cross-sectional imaging with magnetic resonance enterography (MRE), computed tomography enterography (CTE) or ultrasound (US) [73]. These techniques have the advantage of being able to image parts of the bowel that are inaccessible by endoscopy, such as the small bowel. Another advantage of cross-sectional techniques is that they provide an assessment of transmural disease, such as wall thickening and mural edema, and can identify other abdominal or pelvic complications, such as mesenteric venous thrombosis/occlusion, sacroiliitis, pancreatitis, cholelithiasis, or kidney stones [81–84]. A recent analysis of US performance in predicting the need for treatment intensification, which included 89 CD patients, found that mean bowel wall thickness and the presence of the bowel wall Doppler signal were independent predictors of the need for treatment escalation [85].

CTE has a sensitivity of 67% and specificity of 100% for the diagnosis of active CD in the small bowel. Guidelines agree that the use of CT imaging should be minimized because of the long-term risks associated with cumulative radiation exposure [1, 4, 5, 65, 83]. Some authors have suggested that CTE is best suited to elderly patients because of the risk of radiation exposure, and the requirement for fewer breath holds (compared with MRE) [81].

Research suggests that CTE, MRE and small bowel follow-through (SBFT) are similarly accurate for the diagnosis of active CD in the small bowel, but both CTE and MRE show better interobserver agreement than SBFT, and are better at detecting extraintestinal complications [84]. Consensus recommendations from the Society of Abdominal Radiology (SAR) suggest when each type of enterography may be best utilized (Supplementary Table 7) [83]. These SAR guidelines also describe the features of CD on MRE or CTE imaging and how these features should be reported by radiologists to gastroenterologists [83], but they do not make a specific recommendation that one type of imaging is preferable to another or that any particular scoring system should be used. A list of advantages and disadvantages of endoscopic and imaging techniques are shown in Table 1 [72–74, 81, 82, 86–91].

**Table 1:** Advantages and disadvantages of endoscopic and imaging techniques as reported in the published literature [72–74, 81, 82, 86–91]

Advantages	Disadvantages
*Ileocolonoscopy*	
Directly evaluates mucosal healing Possible to take mucosal sample for histology Validated scores of severity (CDEIS, SES-CD, Rutgeerts) Predicts risk of relapse, refractoriness to medical therapy, surgery, postoperative recurrence	Invasive Requires bowel preparation Costly in some countries No transmural evaluation possible Limited small bowel assessment/no visualization of the proximal ileum Controversial definition of mucosal healing (partial versus complete)
*Balloon-assisted endoscopy*	
Allows complete evaluation of the small bowel Directly evaluates mucosal healing Possible to combine with selective small bowel enteroclysis Possible to take mucosal sample for histology Predicts risk of relapse, refractoriness to medical therapy, surgery, postoperative recurrence Provides opportunity for endoscopic therapy (dilatation)	Invasive Low accessibility Requires bowel preparation (when retrograde approach is used) Requires experience and technical expertise Costly No transmural evaluation
*Small bowel capsule endoscopy*	
Complete visualization of the small bowel mucosa Less invasive than conventional endoscopy High diagnostic yield for small mucosal lesions Validated scores of severity (Niv and Lewis scores)	Severity scores need further validation in larger cohorts of patients No ability to sample mucosa for histology Costly Bowel preparation may be required No transmural evaluation Not widely available Risk of capsule retention Usually requires confirmation of GI tract patency (using a patency capsule) before capsule endoscopy in patients with suspected or confirmed CD
*MR enterography*	
Can evaluate the entire bowel tract, including perianal evaluation Assessment of transmural and extramural activity Validated scores of activity (MaRIA, CDMI) No radiation Easily detect strictures, abscesses and fistulas Accurate assessment of healing and prognosis	More expensive than CT or US Time consuming Not widely available Requires bowel distension with oral and/or rectal contrast agent Requires intravenous contrast medium Not suitable for claustrophobic patients or those with sensitivity to contrast agents Low sensitivity for small bowel stricture, and it depends on the degree of small bowel distention
*CT enterography*	
Evaluation of small bowel and colon Evaluation of extraintestinal complications Assessment of transmural and extramural activity Widely available	Radiation exposure No validated scores Requires bowel distension with oral and/or rectal contrast agent Requires intravenous contrast medium Relatively low sensitivity for small bowel strictures, and it depends on the degree of small bowel distention Not enough data regarding the ability of CTE to detect small bowel stricture
*Bowel ultrasound (US)*	
Evaluation of terminal ileum and colon Assessment of transmural and extramural activity No radiation Noninvasive and well-tolerated Widely used Low cost Possible to use Doppler or contrast-enhanced techniques Accurately identifies stenosis, abscesses and fistulas	Limited assessment of proximal ileum, jejunum, transverse colon, and rectum Limited by gas-filled bowel or obesity Accuracy is dependent on the experience of the sonologist Score of activity (SLIC) needs further validation Difficult to compare images during follow-up
*Small bowel enteroclysis/enterography*	
Detects exact anatomical location and extent of the lesions Complete visualization and evaluation of small bowel Easily detects strictures and fistulas Low cost	Low sensitivity for inflammatory lesions Difficult to evaluate mural changes Radiation exposure Accuracy is dependent on the experience of the gastroenterologist Time consuming No validated scores

*CDEIS* Crohn’s disease endoscopic index of severity, *CDMI* Crohn’s disease MRI index, *CT* computed tomography, *MaRIA* magnetic resonance index of activity, *MR* magnetic resonance, *SES-CD* simple endoscopic score for Crohn’s disease, *SLIC* sonographic lesion index for Crohn’s disease, *US* ultrasound

Treatment escalation based on close endoscopic monitoring (> 1 endoscopy within 26 weeks) is associated with an increased probability of mucosal healing (compared with less frequent endoscopic monitoring) [13]. UK guidelines note that the choice of monitoring modality (inflammatory biomarkers, endoscopy, imaging) and the interval between assessments should be individualized based on the site, distribution and severity of the patient’s CD, and patient preference [1]. For example, disease in the upper gastrointestinal tract or proximal small bowel is a predictor of relapse, while inflammation in the terminal ileum is a risk factor for stricturing/penetrating disease (see Sect. 3). Similarly, disease in the colon is a risk factor for perianal disease, which is a poor prognostic indicator in itself (see Sect. 2) [92].

**Table:** 

Statement 1.10: MRE is as accurate as CTE for assessing the severity of intestinal inflammation in patients with CD (Evidence level 1a)	Voting agreement rate 46/47 (97.9%)

A meta-analysis of data from studies using cross-sectional imaging indicates that CTE and MRE are highly accurate for grading CD severity, but US is less so (Supplementary Table 8) [93]. Contrast-enhanced US (CEUS) has greater diagnostic accuracy than standard US (90–95% for CEUS vs 69–88% for wall thickness and/or color Doppler flow on US) [94].

MRE and CTE appear to have similar sensitivity and specificity for detecting active inflammation [1], including in the small bowel [75, 84], although a comparative UK study suggests that MRE may have greater specificity in the assessment of small bowel disease (extent and severity).

**Table:** 

Statement 1.11: Both the CDEIS and the SES-CD are reliable. SES-CD is the recommended scoring system for endoscopic evaluation of intestinal inflammation, based on its validity and simplicity (Evidence level 2a)	Voting agreement rate 46/47 (97.9%)

The most common scoring systems for endoscopic activity in CD are the Crohn’s disease Endoscopic Index of Severity (CDEIS; Supplementary Table 9) [95], and Simplified Endoscopic activity Score for Crohn’s disease (SES-CD; Supplementary Table 10) [96]. The CDEIS produces a score of between 0 and 44, while the SES-CD produces a score of between 0 and 56 [97].

The CDEIS is widely used in clinical trials, but is complex to calculate and requires training/experience to accurately estimate the extent of diseased mucosal surfaces and distinguish between superficial and deep lesions [81]. The SES-CD was developed to overcome these limitations and develop a scoring system more suited to everyday clinical practice [81]. SES-CD scores are highly correlated with CDEIS scores (*r* = 0.938; *p* < 0.0001) [98]; both scores are highly reproducible and show good inter-observer consistency [97, 98]. However, in contrast to the CDEIS, the SES-CD does not take into account the number of explored segments and characterizes ulcers by size rather than by depth [97].

Both the CDEIS and the SES-CD are reliable and validated instruments for quantifying the severity of mucosal inflammation [97]. SES-CD shows slightly better correlation with histopathological scores of disease severity compared with the CDEIS (SES-CD: *r* = 0.600 and CDEIS: *r* = 0.554). However, as described earlier, there is poor correlation between clinical indices of disease activity, such as the CDAI and endoscopic indices; *r* = 0.254 for CDEIS and CDAI, *r* = 0.267 for CDEIS and HBI, *r* = 0.235 for SES-CD and CDAI, and *r* = 0.204 for SES-CD and HBI [99]. Based on the simplicity/ease of use of the SES-CD and the better correlation with histopathology, SES-CD is recommended as the endoscopic index of choice in the current statements.

To date, no threshold levels for either scoring system have been defined as a measure of response or non-response [81, 97, 100]. Our search identified several studies that used the CDEIS and SES-CD indices for the assessment of mucosal inflammation published since 2014, but none used them to make treatment escalation decisions. In all studies, the indices were used for assessing treatment outcomes after escalation [14, 26, 36, 41, 55]. The Organization for the Study of Inflammatory Bowel Disease (IOIBD) used a Delphi process to define such thresholds, and ranked the following as their top 4 definitions of endoscopic remission [97]:

1. SES-CD of 0 to 2
2. CDEIS < 6 (endoscopic remission)/CDEIS < 3 (complete endoscopic remission)
3. Absence of ulceration
4. CDEIS score of ≤ 4.

In STRIDE II, the target for endoscopic remission in CD have been proposed as follows:

1. SES-CD (ulcer subscores = 0 (including aphthous ulcerations) < 3
2. CDEIS (no ulcers) < 3.

Other authors have defined thresholds for the severity of mucosal inflammation (Supplementary Table 11), but these thresholds were arbitrarily set [99]. What is not clear from the literature is whether the absence of remission (based on these scores) should be an indication for treatment escalation. The European Crohn’s and Colitis Organisation (ECCO) guidelines suggest that SES-CD > 6 is indicative of need to escalate treatment to biologic therapy [9]. Based on the data and these recommendations, it would appear that an SES-CD score > 6 during treatment indicates moderate to severe CD which requires treatment escalation.

Histologic assessment of a biopsy sample is not needed to make decisions about treatment escalation. A 2017 Cochrane review noted that there was no uniform methodology for biopsy collection (site) or handling (fixation, sectioning), and none of the available histologic scoring indices has been fully validated [101]. Similarly, STRIDE guidelines (both I and II) note that histologic remission is an important adjunctive assessment measure but should not be a treatment target, because there is insufficient evidence about its clinical value [2, 10].

Histologic healing as a therapeutic endpoint in CD remains challenging. To date, no consensus has been reached to define histologic remission, and none of the 14 different numerical indices been fully validated in all four operating characteristics of reliability, validity, responsiveness and feasibility.

**Table:** 

Statement 1.12: Symptomatic patients: Most patients with worsening signs or symptoms of CD plus objective markers of inflammation (elevated serum level of CRP or leucine-rich α2 glycoprotein (LRG) and/or fecal calprotectin [FC] level) are candidates for treatment escalation, before confirmation of intestinal inflammation by endoscopy or imaging. Patients with fever or abdominal pain should undergo imaging studies to rule out abscess formation. (Evidence level 1a)	Voting agreement rate 37/45 (82.2%)

The STRIDE II guidelines noted “serum and fecal biomarkers are endorsed as intermediate medium-term feasible treatment goals, meaning that at times treatment could be revisited solely based on these tests, to facilitate care in the clinic setting. Elevated serum or fecal biomarkers at times may suffice to revise treatment and at other times require endoscopic confirmation to document the extent and severity of the disease prior to major treatment changes” [10]. Therefore, treatment intensification can be considered in patients with high CRP or FC values and confirmation with endoscopy may also be necessary. However, final endoscopic confirmation is usually needed to assess the achievement of the treatment target of endoscopic remission. The exception to this recommendation is the patient with fever, who should be assessed for the presence of intestinal abscess.

Levels of CRP, FC, LRG and stool lactoferrin (SL) are useful noninvasive markers of inflammation, although FC appears a stronger predictor of relapse in patients with ulcerative colitis than in patients with CD [102, 103]. CRP is a nonspecific marker of inflammation, whereas FC and SL reflect leukocyte trafficking in the gut and are specific to intestinal inflammation [104]. A meta-analysis of studies defined the diagnostic accuracy of these biomarkers for endoscopically active disease (Table 2) [104]. Both CRP levels and FC levels significantly correlate with endoscopic scores of inflammation [105]. LRG levels correlate significantly with the CDAI score in Japanese patients with CD (*r* = 0.361; *p* = 0.044), whereas CRP levels do not (*r* = 0.150; *p* = 0.405) [103], and the correlation between CDAI score and FC is of borderline significance in Japanese patients with CD (*r* = 0.283; *p* = 0.0565) [106]. The sensitivity of FC is greater than that of CRP for detecting mild mucosal inflammation, while CRP appears to be a better biomarker of inflammation in patients with severe systemic inflammation [107]. Typically, in the literature, the threshold CRP level is ≥ 5 mg/L and FC level is > 250 μg/g when these biomarkers are used to define criteria for treatment escalation in CD patients [12, 14, 23, 33, 43, 55, 108]. An SL level of 7.25 μg/g is defined as the optimal threshold for diagnosing endoscopically active disease [104].

**Table 2:** Diagnostic accuracy of biomarkers for detecting endoscopically active disease [104]

Parameter (95% CI)	CRP in IBD	SL in IBD	FC	
			In IBD	In CD
Sensitivity	0.49 (0.34–0.64)	0.82 (0.73–0.88)	0.88 (0.84–0.90)	0.87 (0.82–0.91)
Specificity	0.92 (0.72–0.98)	0.79 (0.62–0.89)	0.73 (0.66–0.79)	0.67 (0.58–0.75)
Positive likelihood ratio	6.3 (1.9–21.3)	3.8 (2.0–7.5)	3.2 (2.6–4.1)	2.7 (2.1–3.4)
Negative likelihood ratio	0.56 (0.44–0.71)	0.23 (0.14–0.38)	0.17 (0.14–0.21)	0.19 (0.14–0.27)
AUC	0.72 (0.68–0.76)	0.87 (0.84–0.90)	0.89 (0.86–0.91)	0.85 (0.82–0.88)
Diagnostic odds ratio	11 (3–38)	16 (6–48)	19 (13–27)	14 (9–22)

*AUC* area under the curve, *CI* confidence intervals, *CRP* C-reactive protein, *FC* fecal calprotectin, *IBD* inflammatory bowel disease, *SL* stool lactoferrin

**Table:** 

Statement 1.13: Asymptomatic patients: Those with elevated levels of CRP or FC should be considered for assessment of intestinal inflammation using endoscopic or imaging studies (Evidence level 1a/1b)	Voting agreement rate 45/45 (100%)

The CALM study (2018) was a randomized comparison of two treatment escalation strategies: one in which treatment escalation decisions were based on CDAI scores ± prednisone use (the clinical management group) and the other in which treatment escalation was based on CDAI scores, elevated levels of CRP (≥ 5 mg/L) and FC (≥ 250 μg/g), and prednisone use [14]. Treatment escalation steps were undertaken at 12-week intervals in patients who met the defined criteria in each group and consisted of (1) starting adalimumab administered every 2 weeks, (2) then weekly, (3) then adding azathioprine. De-escalation could be undertaken at weeks 24 and 36 in patients who did not meet the criteria for treatment failure. Significantly more patients in the tight-control group met the primary endpoint of mucosal healing on endoscopy at week 48 compared with patients whose treatment was escalated in response only to disease activity scores [14]. Other endpoints (including deep remission) also occurred at a significantly higher rate in the tight-control group than the clinical management group. A recently published post hoc analysis of CALM showed that FC < 250 μg/g is useful for predicting endoscopic mucosal healing in CD patients [109].

A systematic review of treat-to-target approaches supports the results of the CALM study, i.e., that treatment escalation based on biomarker evidence of inflammation and signs/symptoms of disease activity is associated with better outcomes than escalation based on signs/symptoms alone [13]. However, more recent individual studies continue to provide conflicting perspectives. For example, a recent Japanese study of 46 CD patients followed up for 1 year following diminished infliximab effects after therapy intensification found that outcomes were improved in patients who received infliximab treatment intensification based on endoscopic findings of exacerbations to a greater extent than in patients monitored via clinical symptoms [110]. On the other hand, the recent STARDUST trial compared a treat-to-target strategy involving early endoscopy, regular biomarker and clinical symptom monitoring, with a clinical maintenance strategy in CD patients with moderate-to-severe disease receiving ustekinumab [111]. Results showed that, at week 48, endoscopic response, endoscopic remission, mucosal healing, and clinical remission were not significantly different when comparing the two approaches, underlying a need for ongoing assessment of studies in this area.

Other biomarkers are in development, including serum calprotectin (SC) [25]. SC may overcome some of the limitations of FC (day-to-day variability, effect of storage conditions, low positive predictive value), but has not yet demonstrated a correlation with mucosal healing [25], and has not been used to determine treatment escalation in any study we could identify.

It is also possible that patients without symptoms have ongoing inflammation, so elevated CRP and FC levels in patients without symptoms should prompt an assessment of mucosal inflammation with endoscopy or other imaging modality [2]. Similarly, there is a possibility for active small bowel lesions in asymptomatic patients with normal CRP or FC levels [112].

**Table:** 

Statement 1.14: The choice of monitoring modality (biomarkers, endoscopy, imaging) and the interval between assessments should be individualized based on the site, distribution and severity of the CD, patient preference, local availability of imaging exams/expertise, and cost (Evidence level 5)	Voting agreement rate 45/45 (100%)

As described earlier (see Supplementary Table 7 and Table 1), each method for assessing intestinal inflammation has advantages and disadvantages. As a result, major guidelines recommend that the choice of modality is individualized based on the site, distribution and severity of the patient’s CD, patient preference, local availability of imaging machines, local expertise, and cost [1]. We recommend monitoring biomarker levels every 3 months in patients with CD, even when the disease is clinically inactive.

**Table:** 

Statement 1.15: Measurement of anti-tumor necrosis factor (TNF)-α drug levels and/or antibody titers may be useful to determine the need for switching or intensifying biological therapy in patients with primary or secondary non-response, but is not necessary at routine check-ups in patients in remission (Evidence level 1b, 5)	Voting agreement rate 46/46 (100%)

Pharmacokinetic or pharmacodynamic causes may underlie poor primary or secondary response to treatment with anti-TNFα therapy [16, 113]. There is considerable evidence to support checking trough drug levels in patients with persistent or worsening symptoms during anti-TNFα therapy [12, 22, 40, 43, 45, 55, 56, 114–121]. In patients with a primary non-response in the first 4–6 weeks, low trough levels of anti-TNFα agents are unlikely to be related to anti-biologic antibody formation [16, 113]. Some commentators have suggested that patients with a primary non-response after 14 weeks of treatment should have antibody titers measured to determine the best approach [16]. US and UK guidelines recommend that patients with a loss of response after initial symptomatic improvement should have drug levels and antibody levels measured [1, 122], but ECCO guidelines do not recommend this approach; because the evidence to support it is weak [9]. The presence of low drug levels and absent or low antibody titers may be an indication to increase the dose of the biological or shorten the dosing interval [113]. Indeed, an analysis of the DIAMOND study comparing adalimumab with adalimumab plus azathioprine in immunosuppressant-naive CD patients found a significant difference in trough levels of adalimumab in patients with and without clinical remission [123]. Further analysis suggested the significance of therapeutic drug monitoring including both trough levels and 6-thioguanune nucleotide levels when managing CD patients receiving combination therapy and independent associations between female sex, increased body and low adalimumab trough levels. If there is evidence of a high antibody titer, or if drug levels are high and antibody titers are absent or low, then a switch to a different biologic agent may be indicated [113]. Using antibody titers and drug levels to guide treatment decisions, in addition to symptoms, does not necessarily improve outcomes compared with using symptoms alone to guide treatment intensification [55, 56], but it does reduce costs [40, 56].

There is some evidence from randomized controlled trials and retrospective observations of real-world clinical practice to suggest that therapeutic drug monitoring (e.g., measuring anti-TNFα blood levels at each maintenance infusion) can improve outcomes or delay the need for surgery in adults and children with CD [12, 43, 124]. However, there are no currently agreed target thresholds for trough anti-TNFα levels and the comprehensive UK treatment guidelines do not advocate therapeutic drug monitoring in the absence of signs/symptoms of active disease [1]. Currently available evidence/expert opinion favors the measurement of drug levels in a reactive manner, i.e., when there are signs of increased disease activity, rather than in a proactive manner, i.e., at each routine check-up [125, 126]. However, when patients have sub-therapeutic drug levels in association with a low titer of anti-drug antibody, optimizing immunomodulator therapy or increasing the dose of anti-TNFα therapy may help to avoid future relapse [125].

**Table:** 

Statement 1.16: Asian patients with CD should be tested for the NUDT15 p.Arg139Cys genotype before initiating thiopurine treatment (Evidence level 4–5)	Voting agreement rate 45/46 (97.8%)
Statement 1.17: When thiopurine therapy is insufficient, consider optimization by increasing the dose (Evidence level 4–5)	41/41 (100%)

Asian patients require lower doses of thiopurines to achieve therapeutically effective blood levels than Caucasians do, and are more susceptible to certain adverse events, such as alopecia and leukopenia [127]. Thiopurine metabolite blood levels are determined by metabolic enzymes, which are influenced by pharmacogenetic factors [127]. Low levels of particular thiopurine metabolites usually reflect genetic variants, which can help determine whether a patient may benefit from a dose increase, whereas a therapeutic/normal level in a non-responsive patient is an indication to switch treatment [1]. The major metabolites affected by genetic variants are the 6-thioguanine nucleotides (6-TGN) [127].

In Caucasian individuals, variations in thiopurine metabolism are mainly due to polymorphisms in the gene for thiopurine S-methyl transferase (TMPT) [1, 127]. However, this is not the case in Japanese patients who show low TMPT activity and a very low prevalence of *TMPT* mutations [127, 128]. In Japan and other east Asian countries, variants in the gene for nucleoside diphosphate-linked moiety X-type motif 15 (*NUDT15*) are a common reason for variation in response and tolerability of thiopurines [127, 129]. *NUDT15* variants reduce the conversion of active thiopurine metabolites to inactive metabolites, leading to higher levels of active metabolites and an increased risk of developing adverse events. Currently, the p.Arg139Cys genotype of *NUDT15* is probably the most relevant pharmacogenetic test for Japanese patients with CD [129]. Approximately 1.1% of the Japanese population has the Cys/Cys genotype of p.Arg139Cys [127]. Based on the profile of enzymes affected by *NUDT15* mutations, the most relevant metabolite to measure in Japanese patients on thiopurines is DNA-incorporated thioguanine (DNA-TG) [127]. However, a recent study Japanese study found that a simple high-resolution melt analysis technique correctly identified the main *NUDT15* genotypes in 1236 of 1241 cases [130].

Another less important mutation that affects thiopurine metabolism in Asian individuals occurs in the *ITPA* gene (specifically a 94C>A single nucleotide polymorphism), which appears to predict the development of adverse effects [127]. The metabolites affected by *ITPA* variants are the TGN, the same metabolites affected by *TMPT* mutations in Caucasians [127].

### Question 2. What are the criteria for treatment intensification in patients with CD with perianal or fistulizing disease?

Target for perianal lesions: healing.

Target for fistula: complete closure.

**Table:** 

Statement 2.1: In patients with CD, perianal disease is associated with numerous poor outcomes and a greater risk of disease relapse prompting a lower threshold for treatment intensification (Evidence level 2c, 5)	Voting agreement rate 45/45 (100%)

Perianal disease is associated with numerous poor outcomes, including high risks of complicated disease course, suboptimal response to therapy or relapse, need for surgery and stoma creation, and hospitalization [131–136]. Diagnostically, perianal lesions may be the first presentation of CD and patients presenting with perianal lesions suspected to be related to CD require evaluation of the bowel. Colonic disease location and penetrating behavior appear associated with the development of perianal disease [131, 136]. In particular, perianal disease appears to be most strongly associated with rectal involvement, having previously been noted in approximately 90% of such patients [137]. In patients with active perianal disease, numerous factors, including complex fistulizing disease, a history of abscess, antibiotic treatment, colonic involvement, smoking, and stricturing phenotype, have been identified that predict poor treatment response [134]. Discontinuation of anti-TNFα therapy in patients who had previously experienced benefit from anti-TNFα therapy, and stricturing phenotype have been associated with a greater risk of relapse in patients with perianal fistulas treated with infliximab or adalimumab [138]. Furthermore, perianal disease is associated with significant impairment in quality of life, especially related to physical symptoms and fecal incontinence [139, 140]. Consequently, perianal disease represents a prompt for intensification of treatment reflected by the fact that the proportion of patients who receive immunomodulator therapy and/or anti-TNFα agents has been shown to increase after the diagnosis of perianal disease [135, 136].

**Table:** 

Statement 2.2: Perianal CD is best managed by a multidisciplinary team including surgeons, coloproctologists, and specialist gastroenterologists (Evidence level 5)	Voting agreement rate 46/47 (97.9%)

Because fistula management is complex and perianal lesions can be the first presentation of CD, treatment decisions should be made in a multidisciplinary context with input from specialist gastroenterologists, surgeons, coloproctologists, and other medical staff [1, 141]. Consideration should also be given to the patient’s potential sense of embarrassment in relation to their symptoms and the impact of complex perianal fistulas on different aspects of life, including relationships, social life and work/professional life.

**Table:** 

Statement 2.3: Initial assessment of the type, extent, and severity of perianal disease as well as the determination of treatment goals is crucial to guiding treatment strategy, both in general and in relation to treatment intensification (Evidence level 5)	Voting agreement rate 46/46 (100%)

Perianal disease may include fistulizing lesions (fistulas and abscesses) and non-fistulizing lesions (ulceration, fissures, and strictures/stenosis) [142, 143]. Various classification systems have been developed to describe perianal disease both in terms of pathological type, anatomical extent, and severity.

The Parks classification of perianal fistulas is well-known but is not specific to perianal CD or suitable for the routine clinical management of perianal CD [144]. Rather, a practical way to categorize fistulas is as simple and complex. Simple fistulas are superficial, or low inter- or trans-sphincteric fistulas with a single internal opening and no purulent reservoirs, rectovaginal fistula, or anorectal stricture [131]. Complex fistulas occur at higher levels, with more than one opening, and often in association with abscesses, anal stenosis or other disease (Supplementary Fig. 1, Table 3).

**Table 3:** Key differences between simple and complex fistula

	Simple fistula	Complex fistula
Type of fistula	Superficial Lower inter- or trans-sphincteric	High inter- or trans-sphincteric, supra- or extra-sphincteric
External opening	Single	Multiple
Perianal abscess	Not present	Present
Vaginal or urethra–bladder fistula	Not present	Present
Rectal stenosis	Not present	Present

Because fistulotomy is associated with nonhealing wounds and a subsequent high rate of proctectomy or incontinence in patients with CD, the American Gastroenterological Association Technical Review on Perianal CD issued in 2003 has recommended that fistulotomy is relatively contraindicated for simple fistula with rectal inflammation or for complex fistula [145]. Rather, it should be indicated only for simple fistula with a single external opening without rectal inflammation.

A previous classification for perianal CD (the Cardiff classification) grades ulceration, fistulas, and strictures on a severity scale of 0 to 2 and classifies fistulas as low or high (Supplementary Table 12) [145].

Malignancy, such as rectal or anal carcinoma, should be excluded at the earliest point of assessment. Determining the extent and presence of fistula openings, their course and the presence of fluid or pus collections is critical when planning treatment [131]. Ulceration, when present, may consist of superficial fissures or cavitating ulcers involving the anal canal or lower rectum with possible extension to perianal skin (aggressive ulceration). Strictures are usually irreversible, leading to anal stenosis or extra-rectal stricture, with severe impact but may in some cases be reversible when physically expanded. The presence of any ulceration and/or stricture in the rectum (proctitis) or inflammation and/or stricture of the anal canal is an important component for fistula assessment [146]. A perianal abscess is clinically defined as fluctuation and radiologically defined as a confined fluid collection and is important to detect during assessment as their timely management minimizes the risk of further septic complications [146].

Fistula activity should be assessed using grading scales, such as the Perianal Disease Activity Index (PDAI) [147], which assesses both disease severity and response to therapy based on measures of quality of life (pain/restriction of activities, and restriction of social activities) and disease severity (fistula discharge, type of perianal disease, and degree of induration). The original description of the PDAI details restriction of sexual activity, although restriction of social activity may be used when these scales are collected insufficiently.

**Table:** 

Statement 2.4: Treatment goals should be determined according to the condition of individual patients with the primary aim of complete closure and, if not possible or expected, the secondary aim of symptomatic relief (Evidence level 5)	Voting agreement rate 46/46 (100%)

Treatment goals for each patient should be based on an assessment of the type, extent, and severity of perianal disease as previously described. In general, the primary aim for perianal fistulas is complete fistula closure. Reported fistula closure rates with various therapies are described in more detail in the discussion of statement 2.6. However, studies reveal that complete closure is frequently not achievable in all patients even with combined medical and surgical therapy. In such patients, it is recommended to pursue the secondary aims of symptomatic improvement or resolution, improvement in quality of life, and prevention of septic complications. Ultimately, the long-term goal of treatment is to maintain anal function.

In terms of achieving treatment goals, history and local physical examination is well-suited to monitoring discharge, local symptoms, and restrictions on physical and sexual or social activity. On the other hand, objective imaging studies can more easily assess the location, distribution, activity, abscess formation, type of fistula and factors that may be contributing to symptoms and signs (e.g., collections, stricturing). Consensus guidelines from France have outlined targets to be attained, such that treatment intensification is not required, in the domains of symptoms, physical examination findings, and MRI findings [148].

**Table:** 

Statement 2.5: The sequence and choice of treatment strategy, including surgical procedures and use of anti-TNFα therapy, should be based on stepwise assessment of the lesion(s) and response to treatment (Evidence level 1, 5)	Voting agreement rate 45/45 (100%)

**Fig. 1:**
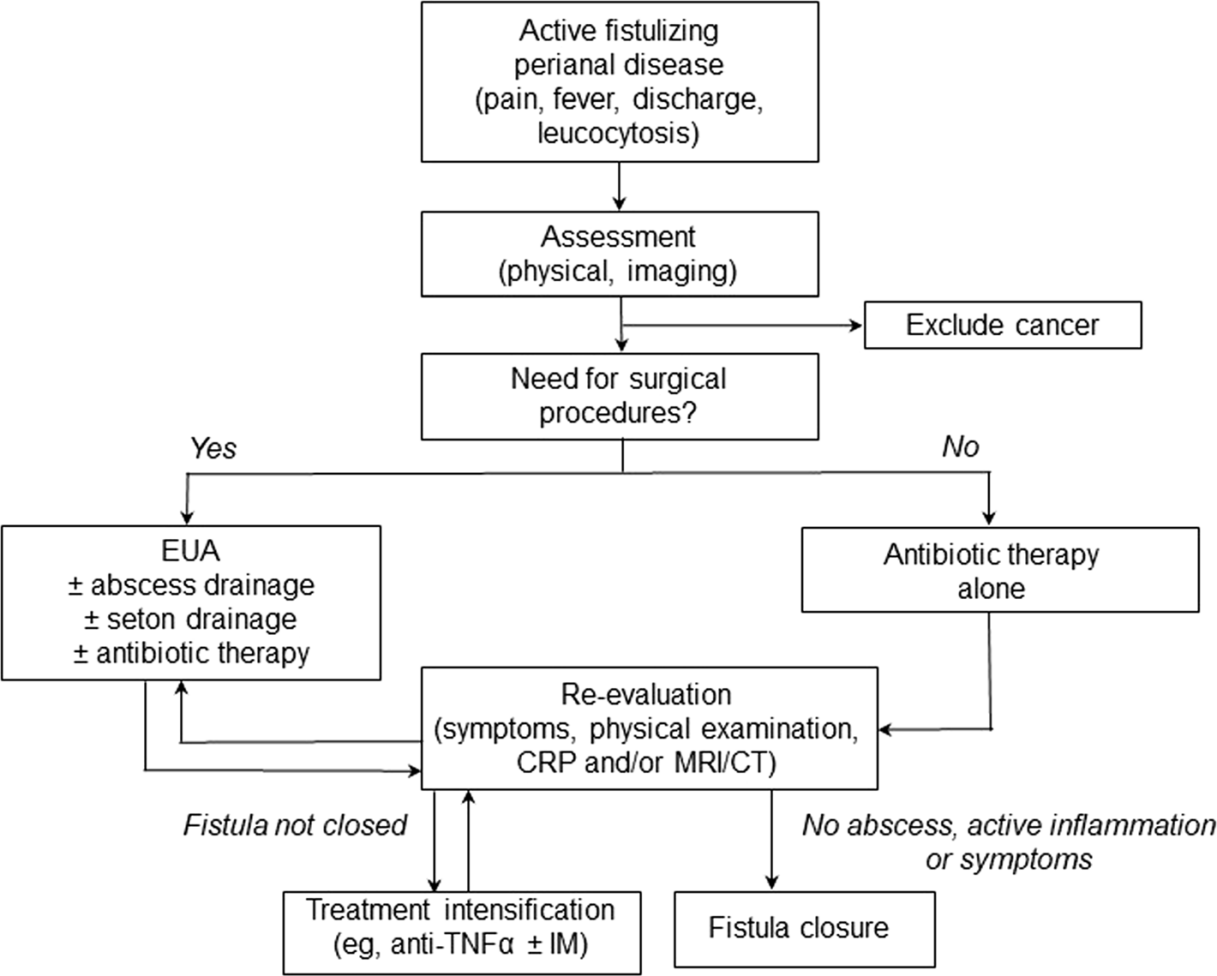
Initial assessment and choice of therapy in patients with active fistulizing perianal disease. *CRP* C-reactive protein, *CT* computed tomography, *EUA* examination under anesthesia, *IM* immunomodulator, *MRI* magnetic resonance imaging, *TNF* tumor necrosis factor

In patients with active fistulizing perianal disease, the choice of treatment strategy should be based on a stepwise assessment of the lesion(s) and their response to treatment as outlined in Fig. 1.

Patients with suspected coexisting abscesses should be evaluated early by surgical consultation and examination under anesthesia (EUA) for possible drainage and/or seton insertion. Antibiotics, especially metronidazole and ciprofloxacin, are commonly used and recommended for initial symptom control. Although antibiotics alone may reduce fistula drainage and achieve fistula closure in some patients, this response may not always be sustained after cessation [144, 149]. Fistula closure rates of 20–50% have been noted with metronidazole but recurrence rates of up to 80% have also been noted following cessation [149]. As a result, antibiotics have been viewed as a bridge to more definitive treatment strategies [144]. Imaging studies, including proctoscopy, proctosigmoidoscopy, and pelvic MRI are strongly recommended to delineate the anatomy of the fistula tract(s) and determine if complicated or uncomplicated fistulizing disease is present. CT may also be used, where other options are not readily available, although the diagnostic accuracy of CT is lower than that of MRI. However, initial assessment should not be restricted to pelvic MRI or CT but also include evaluation for the presence of colon and rectal lesions. Patients with complicated disease are generally referred initially for surgical consultation, whereas those with uncomplicated disease can be initially treated with medical therapy. For the closure of perianal fistulas, both infliximab and adalimumab have been shown to be effective in a number of studies [138, 150–155], and are recognized as the standard of treatment to induce symptomatic response and complete closure [144, 146]. Consensus statements also suggest that anti-TNFα therapy is combined with immunomodulator therapy (thiopurines), again based on low-quality evidence, to increase the chances of fistula closure and long-term remission (see statement 2.6 for study details) [144]. Optimization of anti-TNFα therapy by measurement of trough concentrations and assessment of anti-drug antibodies may improve outcomes in patients with perianal disease. Observational studies have shown that higher trough levels of anti-TNFα therapy are associated with more favorable fistula response and lower rates of relapse in patients with perianal fistula [156–159]. Although trough measurement is not indicated in Japan, consideration should be given to increasing the trough concentration by increasing the dose of anti-TNFα therapy or shortening the administration period in cases of insufficient therapeutic response.

The extent of fistula closure should be assessed throughout these steps to guide treatment decisions. Recent guidelines have defined response and remission to treatment in patients with fistulizing, perianal CD as follows [144]:

- Symptomatic response: Meaningful improvement in symptoms of pain and discharge as judged by both the patient and physician in the absence of remission. As noted previously, symptomatic response should not be considered the primary aim of treatment. However, symptomatic response is useful to assess early treatment response and may be a secondary aim if the primary aim of closure is not achievable. The degree of symptomatic response can be determined by reference to the PDAI (Supplementary Table 13). Reduction in the total score by ≥ 4 points suggests meaningful recovery, whereas a reduction of ≤ 3 points suggests no change or possible exacerbation of disease [145].
- Symptomatic remission: This is marked by absence of both pain and discharge from the fistula tract. In terms of the PDAI scoring system, remission would be characterized by a score of 0 in the items related to discharge, pain and restriction of activities, and possibly restriction of sexual activity.
- Radiographic response: Severity of MRI findings can be quantified using scoring systems, such as the van Assche score, which provides specific subscores in the domains: number of fistula tracks, location, extension, hyperintensity on T2-weighted images, collections (> 3 mm diameter) and rectal wall involvement [160]. Van Assche scores > 15 have been associated with significantly lower long-term healing rates in a prospective, observational study of 70 patients with CD and anal fistulas [161]. A similar but more recently developed scoring index (MAGNIFI-CD) based on 6 items also determines perianal fistulizing activity but with improved operating characteristics compared with previous systems [162]. Accurate assessment of the number, location and extent of tracts, especially if multiple, may be difficult. However, hyperintensity on T2-weighted images, presence of collections and rectal wall involvement in terms of thickening are more readily assessable.
- Radiographic remission: In conjunction with use of the radiographic scoring systems described above, true radiographic remission can be viewed simply as the absence of inflammation in any fistula tract and the absence of any abscess.
- Complete remission: This can be viewed as the combined presence of symptomatic and radiographic remission.

Response and remission can be determined by fistula drainage assessment such as that used in clinical trials, which defined improvement as a decrease from baseline of ≥ 50% in the number of open draining fistula for at least two consecutive visits (≥ 4 weeks) and remission as closure of all fistula that were draining at baseline for at least two consecutive visits (≥ 4 weeks) [163]. However, such strict definitions may be difficult to apply in routine practice, where the close observation periods of clinical trials may not be practically feasible. In such patients, clinicians should also consider predictors of poor outcomes in patients with perianal fistulizing disease, including complex fistulas, colonic location, stricturing phenotype, and a history of perianal abscess [134, 138, 164].

**Table:** 

Statement 2.6: Treatment intensification in patients with perianal disease should be guided by the nature of the lesion(s) and response to initial therapy (Evidence level 1, 5)	Voting agreement rate 36/37 (97.3%)

Treatment intensification in patients with perianal disease should be based on the treat to target principle with the aim of optimizing therapy to achieve fistula closure according to physical examination findings and objective diagnostic imaging studies. However, treatment intensification may not achieve this aim, in which case secondary goals, such as those in relation to symptomatic and functional improvement, should be set.

Assessment of actual and likely response is crucial when considering treatment intensification. Prognostic factors for greater chance of successful healing include the absence of proctitis, short duration of fistulizing disease, nonsmoking status, and simple fistula [165]. Complex fistulas, on the other hand, predict a worse response [164, 166]. As during the initial phase of treatment, inadequate symptomatic response or loss of symptomatic response should prompt consideration of repeated surgical management to clarify the need for surgery before intensifying treatment. If surgery is deemed necessary, surgery should be performed first before simply intensifying medical treatment. However, fistula relapse is common following perianal surgery [167], and evidence from at least one retrospective review suggests that drug escalation after initial surgery is associated with a significantly reduced likelihood of reoperation [168]. In patients treated with anti-TNFα therapy who have achieved a symptomatic response, ongoing reassessment is needed to determine if treatment can be maintained at the same level or if the dose of anti-TNFα agent should be increased. Immunomodulator therapy may also be required if this has not already been commenced. In patients who have undergone treatment optimization, ongoing monitoring based on physical examination findings and diagnostic imaging studies should be maintained with the primary aim of fistula closure.

Favorable response to both anti-TNFα therapy and thiopurines, including in terms of closure, have been noted in the literature. A meta-analysis of 5 randomized trials reported a higher rate of fistula response with azathioprine or 6-mercaptopurine than placebo (odds ratio, 3.09 [95% CI 2.45–3.91]) [169]. Complete fistula response (i.e., closure) was higher with 6-mercaptopurine (31%) than placebo (6%) in an early long-term randomized controlled trial of 83 patients [170]. Fistula closure with infliximab 5 mg/kg was also significantly higher than with placebo (55% vs 13%; *p* = 0.001) in a randomized, double-blind trial with a median duration of response of approximately 3 months [153]. The ACCENT II trial confirmed the long-term response of infliximab with a sustained response (in terms of predefined reduction in draining fistulas) noted in twice as many patients treated with infliximab than placebo (46% vs 23%; *p* = 0.001) [155]. Similar results were seen in the CHARM trial, which demonstrated a significantly higher complete fistula closure rate for adalimumab than for placebo after 26 weeks (30% vs 13%; *p* < 0.001) [171].

A practical question in terms of treatment intensification concerns timing of dose increase and dose combination. In the ACCENT II trial involving infliximab monotherapy [155], the peak time to loss of response among patients with a response at randomization was approximately 10 weeks. Hence, a period of 3–6 months seems reasonable to assess initial response to therapy and decide whether further increase in anti-TNFα dose or combination with thiopurine treatment may be necessary. There are limited studies to thoroughly assess the efficacy of anti-TNFα therapy and thiopurines in combination and results appear to be mixed. The rate of fistula response during the open-label phase of the ACCENT II study was identical in patients receiving concomitant immunomodulator therapy and those receiving infliximab monotherapy alone [155]. However, an open-label case series suggested that combination therapy with infliximab and an immunosuppressant (purine analog or methotrexate) was associated with a greater likelihood of first perianal fistula closure (HR 2.58, 95% CI 1.16–5.60) compared with infliximab alone, especially in patients who were naïve to immunosuppressants [172]. These results are supported by those of a prospective observational cohort study of 41 patients with perianal fistula which found that combination anti-TNFα and thiopurine therapy led to clinical benefit (remission or response) in 58% of patients up to the end of the 3-year follow-up period [173]. Despite issues with the evidence for combination therapy, many consensus guidelines recommend the use of a thiopurine in combination with anti-TNFα therapy, including at initiation of therapy.

Treatment intensification strategies are more complex in cases of stenosis. Treatment pathways for anorectal stenosis associated with CD depend on the type of stenosis (fibrous, inflammatory) or presence of dysplasia, which generally necessitates proctectomy regardless of the grade [142, 174]. First-line treatment of an inflammatory anal or rectal stenosis is medical treatment with failure of treatment prompting optimization of therapy or dilatation, which is the first-line strategy for isolated fibrous stenosis [174]. Medical therapy with an anti-TNFα agent and immunomodulator may also be used in combination with surgical drainage and dilatation in patients with concomitant stenosis and anoperineal suppuration [142, 174]. Finally, the possibility of malignancy should be considered throughout the clinical course, especially when there are symptoms such as worsening pain and increased drainage, or when the treatment effect is poor.

### Question 3. What are the criteria for treatment intensification in patients with CD with small bowel stenosis?

Target: Resolution of obstructive symptoms.

**Table:** 

Statement 3.1: Assessment of small bowel stenosis is indicated in CD patients with new onset or worsening of obstructive symptoms, including acute abdominal distension, cramping, nausea, vomiting, and abdominal pain (Evidence level 5)	Voting agreement rate 45/46 (97.8%)

Stenosis is a common complication of CD, particularly in patients with small bowel disease [175], or after surgery [176]. The definition of this condition varies between studies [177], but it is generally accepted to be a narrowing of the bowel lumen caused by thickening of all layers of the bowel wall [177, 178]. Narrowing of the bowel can relate to edema associated with acute inflammation, whereas fibrosing stenosis develops after prolonged inflammation [179]. Therefore, any narrowing can be predominantly inflamed, predominantly fibromatous or mixed [180]. Fibrosis occurs because of the pleiotropic effects of inflammatory mediators [177]. Chronic inflammation stimulates fibroblast activation in the extracellular matrix, leading to collagen deposition, tissue remodeling and stenosis formation [179]. The term stricture is often used to describe fibrotic narrowing, with or without an inflammatory component [83].

Because CD tends to be progressive, the current hypothesis is that the bowel wall remodelling occurs after years of accumulating inflammatory damage, resulting in stenotic complications [177]. However, stenosis may sometimes be present at diagnosis [181]. Therefore, physicians should have a high index of suspicion for small bowel stenosis in patients who present with or develop obstructive symptoms, particularly if they have ileal CD.

The key symptoms of small bowel stenosis are acute abdominal distension, cramping, nausea, vomiting, abdominal pain, which is typically postprandial [182]. Patients with such signs and symptoms should be investigated for the presence of stenosis [1]. Bouhnik and colleagues have developed the CD obstructive score, which incorporates the severity and duration of obstructive pain, the presence and duration of nausea and/or vomiting, whether the patient is voluntarily limiting their diet because of symptoms, and the consequences of obstructive symptoms (e.g., hospitalization) [183]; however, this score is not widely used in clinical practice or in clinical research.

**Table:** 

Statement 3.2: A number of imaging modalities can be used to identify small bowel stenosis, including cross sectional imaging modalities (US, CTE and MRE), small bowel radiography and balloon-assisted endoscopy. If available, MRE is the preferred imaging modality for identifying small bowel stenosis (Evidence level 2b, 4)	Voting agreement rate 42/44 (95.5%)

As described in more detail in statement 1.7, cross-sectional imaging modalities are particularly useful for the assessment of the small bowel [72, 75, 81–84], and are able to detect mural changes associated with stenosis [175]. Endoscopy is particularly good at identifying inflammation on the luminal surface, but endoscopists may not be able to determine whether the narrowing is fibrotic or edematous (inflammatory) [176, 180]. US has the advantage of being non-invasive and allowing interaction with the patient during the procedure, so the patient can direct the sonographer to the areas of greatest discomfort, which can help to detect lesions responsible for the symptoms [176]. However, diagnostic yield may be lower with ultrasound than with magnetic resonance enteroclysis [180].

A recent comprehensive systematic review of the literature by Bettenworth and colleagues on different radiographic modalities including ultrasound, CT, MRE, and hybrid PET was conducted [175]. The reported sensitivity and specificity of these modalities are summarized in Supplementary Table 14 [175, 179].

Based on an analysis of the literature, these authors recommend MRE as the preferred imaging modality based on its accuracy, availability, and lack of exposure to radiation [175]. This recommendation is supported by data from a Japanese study, which found that MRE has 82.8% accuracy for diagnosis of small bowel stenosis, and 87.8% accuracy for the diagnosis of major small bowel stenosis [184]. However, this study [184] also found that MRE has lower sensitivity for detecting small bowel stenosis (58.8% for major stenosis and 40.8% for any stenosis) than the sensitivity reported in the review by Bettenworth et al. (75–100%) [175]. Balloon-assisted endoscopy, in addition to MRE, may be beneficial if the patient has obstructive symptoms as indicated in statement 3.3 [185]. Unfortunately, in patients with stricturing disease, SES-CD based on conventional ileocolonoscopy does not correlate with MaRIA score nor does it predict the need for treatment escalation, [186]. This is likely because the stenosis prevents deep insertion of the scope for many patients, thereby limiting the ability of endoscopy to assess inflammation. A more recent review of MRE over a 5-year period from Australia confirmed that MRE is useful in patients with strictures and was able to predict the need for surgery as well as which patients may benefit from treatment intensification [187].

Contrast enhancement techniques can help differentiate between fibrotic and edematous narrowing [175, 179], but there are limited comparative data to guide a recommendation for one imaging modality over another in this regard. Some studies have suggested that CT modalities have limited ability to detect fibrosis [188].

**Table:** 

Statement 3.3: Even if cross-sectional imaging, including MRE, is negative, balloon-assisted endoscopy is recommended in patients with obstructive symptoms; this allows the option of performing endoscopic balloon dilatation in suitable candidates (Evidence level 2b, 4)	Voting agreement rate 45/46 (97.8%)

Data from Japan indicate that about one in four CD patients with a negative result on MRE will have stricturing present on balloon-assisted endoscopy [185]. In this study, balloon-assisted endoscopy was able to detect stenosis in MRE-negative patients that were less severe than the ones in MRE-positive patients, i.e., stenosis with greater diameter, shorter length, and with a lower incidence of prestenotic dilatation [185]. Importantly, 10.8% of the patients who were MRE-negative but had positive findings on balloon-assisted endoscopy required further surgery [185]. Therefore, we recommend balloon-assisted endoscopy especially for symptomatic patients and those with a negative result on MRE to ensure detection of stenosis. An additional advantage of balloon-assisted endoscopy is that endoscopic balloon dilatation can be performed if a stenosis is identified and the patient is a suitable candidate (see statement 3.5). A recent study from Japan highlighted the role of balloon-assisted enteroscopy for evaluating deep small bowel lesions even in patients in clinical remission [189]. Multivariate logistic regression analysis found that the Harvey–Bradshaw Index and a partial Simple Endoscopic Score for CD independently predicted relapse. The authors concluded that balloon-assisted enteroscopy is important for ongoing evaluation of small bowel lesions even among patients in remission.

**Table:** 

Statement 3.4: Treatment selection in stenotic CD depends on whether the stenosis is primarily inflammatory or fibrotic. Surgical treatment or endoscopic balloon dilatation should be considered for fibrotic-predominant stenosis indicated by clinical features and imaging findings (Evidence level 2a)	Voting agreement rate 39/46 (84.8%)

**Fig. 2:**
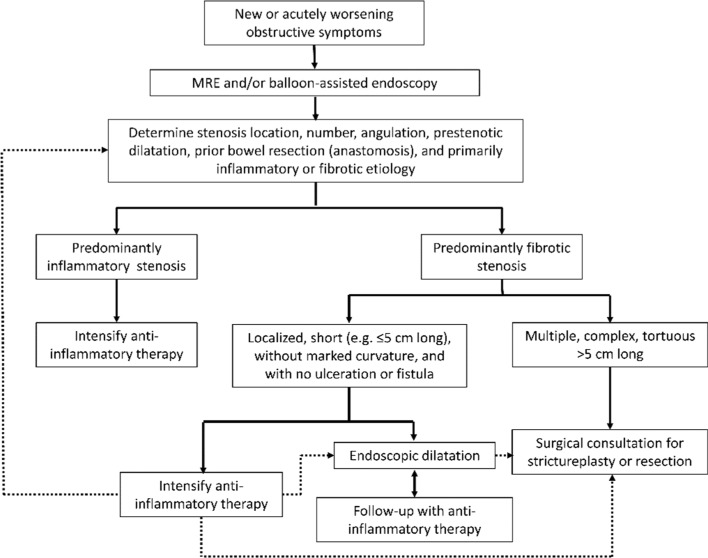
Algorithm for the assessment and management of small bowel stenosis in CD. Dotted lines indicate pathways for patients who show no improvement in obstruction. *MRE* magnetic resonance enterography

Guidelines recognize the importance of distinguishing between predominantly inflammatory and predominantly fibrotic stenosis, when determining treatment approaches in patients with small bowel stenosis [1]. Optimized medical therapy is indicated when the stenosis is predominantly inflammatory, whereas patients with fibrotic stenosis are better managed by surgery or endoscopic balloon dilatation (Fig. 2) [1].

However, differentiating between a predominantly inflammatory and predominantly fibrotic stenosis requires careful consideration of a range of factors including inflammatory biomarkers (such as CRP levels), clinical and imaging features, and response to medical therapy.

While CRP levels can indicate the presence of a spike in inflammatory activity, there is currently no biomarker that can determine which patients have primarily inflammatory vs fibrotic stenosis. The observational CREOLE study identified the following clinical and imaging factors as significantly associated with treatment success (and, therefore, a primarily inflammatory stenosis): severe obstructive symptoms lasting < 5 weeks, moderate prestenotic dilatation (diameter of 18–29 mm) and marked delayed phase enhancement on MRE [183]. The study authors proposed a stenosis scoring system and suggested that the effectiveness of adalimumab varies according to the score. While the CREOLE study was not randomized, it provides some guidance on the patients who are most likely to benefit from a trial of adalimumab. No comparable data were available to determine whether the same criteria could be used to identify suitable patients for treatment with other anti-TNFα therapy.

Obstructive symptoms that do not resolve with intensified medical therapy suggest a fibrotic etiology; patients with such ongoing symptoms should be assessed for a more interventional approach, such as endoscopic balloon dilatation or surgery.

Patients without signs of inflammatory stenosis (defined above) probably have fibrotic stenosis and are unlikely to respond to medical treatment, so immunosuppressive therapy will subject them to adverse events for minimal clinical benefit [183]. These patients may benefit from endoscopic balloon dilatation, strictureplasty or surgical resection [1, 190].

**Table:** 

Statement 3.5: Before endoscopic balloon dilatation therapy, the length, location and angulation of the stenosis should be assessed. Endoscopic balloon dilatation should not be undertaken if fistulas or deep ulcers are present within the stenosis (Evidence level 2a)	Voting agreement rate 46/46 (100%)

Endoscopic balloon dilatation is indicated for a short stenosis (≤ 5 cm) [191–193], that does not respond to medical therapy [178]. Longer, severe, frequently recurring, or more complex stenosis may best be treated with strictureplasty or surgical resection [1, 178, 190].

A recent meta-analysis by Bettenworth and colleagues (2020) examined outcomes of endoscopic balloon dilatation specifically in patients with small bowel stenosis, excluding those with colonic stenosis or stenosis of the ileocolonic anastomosis [194]. Approximately 82% of these patients achieved symptomatic relief after the procedure [194]. Factors significantly associated with increased risk of unsuccessful outcome in patients with small bowel stenosis were endoscopic evidence of active disease in jejunum and/or proximal ileum and Asian race [194]. Irrespective of the site of the stenosis, factors that increase the risk of complications after endoscopic balloon dilatation are a predominantly inflammatory stenosis with a high level of disease activity, multiple stenosis, a tortuous or tethered small bowel or significant stenosis angulation, complete bowel obstruction, adjacent penetrating ulcer or intra-abdominal collection, fistulization within 5 cm of the area to be dilated, or stenosis caused by extrinsic compression (e.g., adhesions) [190, 191]. Contraindications to endoscopic balloon dilatation are stenoses associated with an abscess, phlegmon, fistula, high-grade dysplasia, or malignancy [190].

Two systems are available for endoscopic balloon dilatation: over-the-wire (OTW) balloon catheter and through-the-scope (TTS) balloon but the TTS system tends to be used more frequently [190, 192]. Balloon dilatation can be graded, in which the balloon size is incrementally increased, or non-graded using a single balloon size, but the graded approach appears to be the most common [192], and is predictive of a successful outcome in patients with small bowel stenosis [194]. The balloon catheter can be inserted via an antegrade or retrograde approach. The choice of system, approach (antegrade/retrograde), technique (graded vs non-graded), dilatation time, and size of the balloon should be tailored to each patient according to their anatomical site and features, including luminal diameter and stenosis length [190, 192].

Technical failure (inability to dilate the stenosis) occurs in about 6–11% of patients undergoing endoscopic balloon dilatation of the small bowel [194, 195], and major complications occur in 4–6% [192, 194–196].

**Table:** 

Statement 3.6: Endoscopic balloon dilatation contributes to the avoidance or delay of surgery (Evidence level 2a)	Voting agreement rate 43/45 (95.6%)

Most patients who develop a stenosis will experience a recurrence after endoscopic balloon dilatation and need to undergo a second dilatation or surgery [194, 196]. Data from the only prospective study on outcomes after endoscopic balloon dilatation of a small bowel stenosis in Japan showed that 36% of patients required redilatation at 2 years, and 53% needed redilatation at 3 years [197]. This is lower than the proportion estimated in the meta-analysis by Bettenworth and colleagues; that study estimated that 55.4% of patients require re-dilatation within 2 years [194, 196].

However, data on surgery avoidance were similar in the long-term Japanese study and in the meta-analysis. The long-term Japanese study by Hirai and colleagues reported that 21% of patients who had undergone endoscopic balloon dilatation of a small bowel stenosis underwent surgery within 2 years, and 27% required surgery within 3 years [197]. A nationwide retrospective study from Japan reported surgery-free rates of 74.0% at 1 year, 54.4% at 5 years, and 44.3% at 10 years after endoscopic balloon dilatation of small bowel stenosis [198]. The use of immunomodulators or anti-TNFα therapy after the onset of obstructive symptoms significantly increased the likelihood of avoiding surgery after endoscopic balloon dilatation. Other factors that were associated with being able to avoid surgery were non-stricturing or non-penetrating disease at onset, mild symptoms, successful endoscopic balloon dilatation, and stenosis length < 2 cm [198].

### Question 4. What considerations are essential for treatment intensification in patients with CD in the postoperative setting?

Target: endoscopic remission.

**Table:** 

Statement 4.1: Patients who have undergone surgery for CD are at risk of relapse and reoperation, so postoperative treatment is usually needed. Prior to the scheduled endoscopic assessment after surgery, postoperative treatment decisions should consider preoperative treatment responses and underlying risk factors (i.e., current smoking habit, penetrating disease or previous resection, residual active disease, extensive lesions, perianal disease) (Evidence level 1b, 5)	Voting agreement rate 46/47 (97.9%)

Approximately 10–38% of patients who undergo surgery for CD have symptoms of clinical recurrence, and 35–93% have endoscopic lesions, within 12 months [199]. Endoscopic disease recurrence predates symptomatic recurrence after surgery for CD [200], and clinical disease activity shows poor correlation with endoscopic disease activity after surgery [201]. Therefore, postoperative management should take a more proactive than reactive course.

Before endoscopic assessment at 6–12 months after surgery, treatment decisions should take into account the patient’s overall risk of recurrence (Supplementary Table 15), response to preoperative treatment, and type of surgery [202].

Currently, there is no validated risk score to guide treatment decisions after surgery [203], and the definition of ‘high risk’ differs in the literature (Supplementary Table 15). The POCER study defined high risk by the presence of current smoking, penetrating disease or previous resection [204], whereas French and UK guidelines, and a recent narrative review, require the presence of ≥ 2 risk factors [1, 6, 202]. Other high-risk clinical or endoscopic features for recurrence include extensive small bowel disease, perianal disease, penetrating disease, endoscopically active disease, and the presence of granulomas or myenteric plexitis [1, 6, 202]. An interval of less than 5 years between a first and second surgery for CD is predictive of the need for a third operation [205].

In the postoperative setting, the use of anti-TNFα therapy may reduce the risk of recurrence in patients with active CD [204, 206], and there is evidence to suggest that these agents are superior to aminosalicylates [203]. Longitudinal data show that the risk of reoperation in CD patients has been significantly lower since 2002, when anti-TNFα therapy was introduced in Japan, although the change may also have been influenced by changes in surgical techniques [206].

The POCER study showed that active care with early endoscopy and treatment escalation based on endoscopic recurrence significantly improves outcomes in CD patients after bowel resection [204]. It also demonstrated that a risk-guided approach to therapy is valuable in the postoperative setting, taking into account the length of the patient’s remaining bowel, and the underlying risk factors for recurrence. Smoking was the most important determinant of disease progression (odds ratio for recurrence of 2.4 [95% CI 1.2–4.8]; *p* = 0.02) [204]. Therefore, patients should be strongly advised to quit smoking. In patients who are unable to stop smoking after surgical resection, aggressive postoperative treatment of CD with early escalation of medical therapy is warranted.

**Table:** 

Statement 4.2: CD patients who have undergone intestinal resection should be endoscopically assessed for intestinal inflammation 6–12 months after surgery, irrespective of their risk profile, and treatment decisions should consider this result. Evidence of mucosal inflammation is required for a diagnosis of CD recurrence, using endoscopy, cross-sectional imaging or biomarker tests (Evidence level 1b, 5)	Voting agreement rate 46/48 (95.8%)

**Fig. 3:**
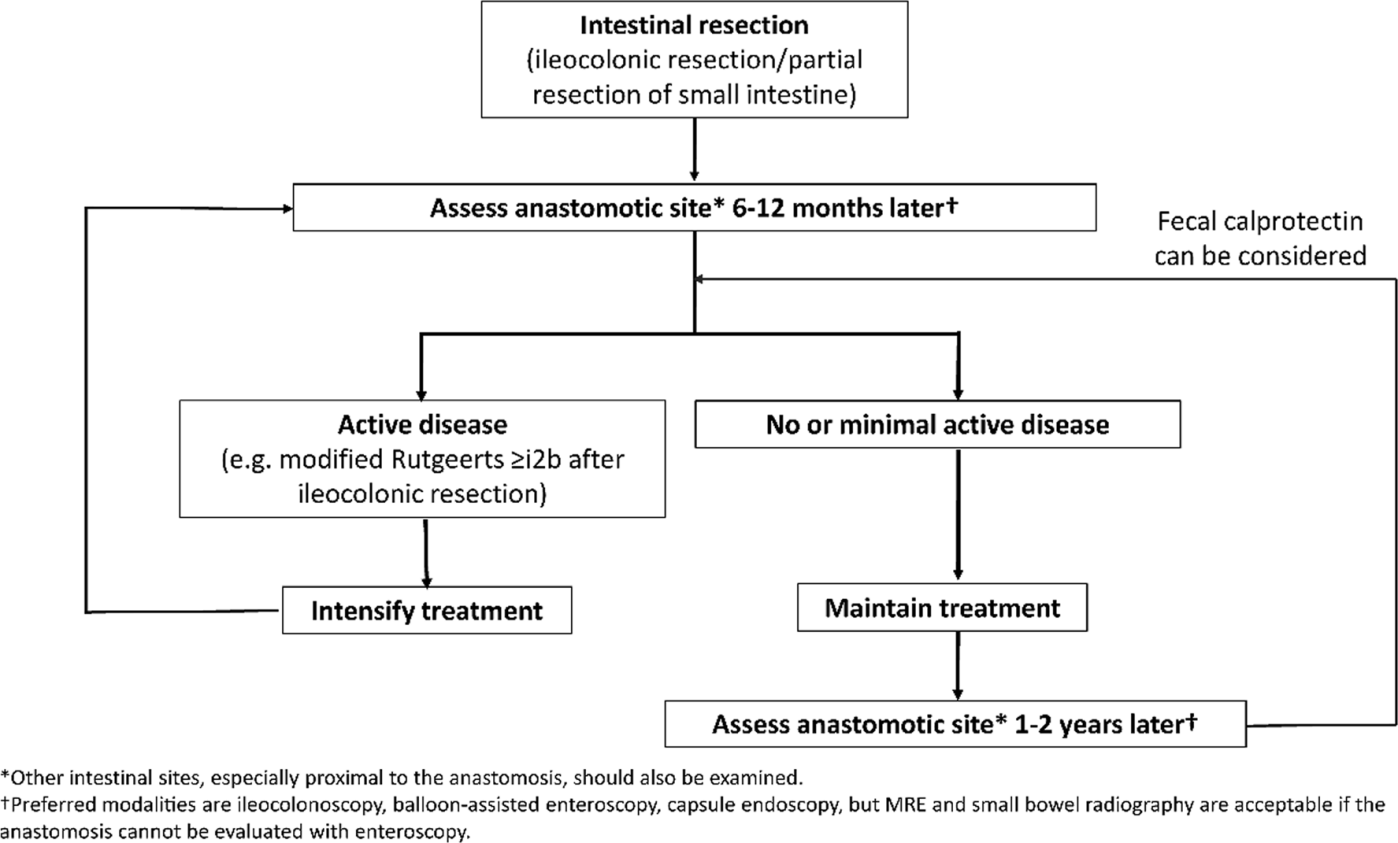
Algorithm for the monitoring and treatment of patients after intestinal resection in Japan

Based on data from the randomized POCER study [204], most international guidelines agree that, after ileocolonic resection, patients at high risk of recurrence should receive treatment with immunomodulators or anti-TNFα therapy [1, 6, 207]. On the other hand, medical management can be guided by endoscopic recurrence in patients who are not at high risk [202, 208]. We recommend that patients who have undergone ileocolonic resection should undergo endoscopic assessment 6–12 months after surgery, irrespective of their risk profile or medical management. Figure 3 shows the recommended approach to postoperative monitoring and treatment in Japan.

**Table:** 

Statement 4.3: Endoscopy is the gold standard for mucosal assessment, but cross-sectional imaging with MRE or US is an alternative if endoscopy is not feasible or appropriate. Balloon-assisted endoscopy or capsule endoscopy are appropriate modalities for small bowel visualization (Evidence level 3a, 5)	Voting agreement rate 47/47 (100%)
Statement 4.4: The anastomotic site and proximal small bowel should be evaluated after intestinal resection. The presence of a modified Rutgeerts score of i2b or higher after ileocolonic resection is indicative of a high risk of clinical recurrence. Treatment escalation should be considered in patients with active inflammation (e.g., a modified Rutgeerts score ≥ i2b) at any time after bowel resection, regardless of the presence or absence of abdominal symptoms (Evidence level 1b, 3b, 5)	Voting agreement rate 43/46 (93.5%)

Direct visualization of the bowel via endoscopic techniques is the gold standard for mucosal assessment after surgery [1, 200]. While visualizing the anastomotic site is important, it is also necessary to assess all areas of the bowel likely to be affected by inflammatory changes. The imaging modality should be chosen according to the site of the patient’s CD and the operative procedure, so that the anastomotic site and proximal small bowel can be visualized. If conventional ileocolonoscopy cannot reach the site of anastomosis or the patient has known small bowel involvement, balloon-assisted endoscopy should be considered instead of, or in addition to, ileocolonoscopy. Balloon-assisted endoscopy is usually undertaken via the anal route, but if this technique is unable to reach the anastomosis, endoscopy can be undertaken using the oral route [71]. Capsule endoscopy is a reasonable approach to visualizing the entire bowel in the postoperative setting [209, 210], and is usually performed after successful passage of a patency capsule.

Small bowel enteroclysis/enterography is frequently used for monitoring in the postoperative setting because of their widespread availability in Japan, and ability to accurately detect mucosal abnormalities, although it carries a risk of radiation exposure [211].

Cross-sectional techniques such as bowel US and MRE do not visualize the mucosal surface, and are, therefore, not the preferred method of postoperative assessment, but they can provide complementary information to endoscopy, particularly in relation to involvement of the bowel wall and adjacent tissues [176]. A systematic review and meta-analysis of data with different imaging modalities suggests that they are all highly sensitive in the detection of postoperative recurrence [212], although the sensitivity of MRE for detecting stenosis is only approximately 40% [184]. Contrast enhancement improves the diagnostic accuracy, sensitivity and specificity of bowel US relative to standard bowel US [212, 213], CT or CTE can be used as an option if bowel US or MRE is not available; however, it is important to minimize the risk of radiation exposure.

Patients require regular monitoring after surgery. Patients without evidence of active CD at the first postoperative assessment should undergo regular clinical evaluation, with endoscopy at least every 2 years [208]. The modified Rutgeerts score (Supplementary Table 16) was developed for endoscopic assessment after ileocolonic resection [214]. A score of i2 or higher is a significant predictor of symptomatic recurrence [215]. Currently, there is no accepted scoring system for endoscopic assessment after other types of surgery. In the absence of an accepted scoring system, we recommend using the modified Rutgeerts score after any type of bowel resection.

**Table:** 

Statement 4.5: The CD activity index (CDAI) and HBI are poorly indicative of endoscopic recurrence after surgery and are not sensitive enough to monitor early postoperative recurrence (Evidence level 3b)	Voting agreement rate 47/47 (100%)

Approximately 50% of patients with CD show symptomatic recurrence within 3 years of surgical resection [199]. Therefore, patients require regular monitoring for signs and symptoms of disease activity. However, the CDAI is not sensitive enough to monitor disease activity in the postoperative setting as it shows poor correlation with endoscopic recurrence (Pearson *r* = 0.07) [216]. As described above, endoscopic monitoring is required after surgery to identify disease recurrence.

**Table:** 

Statement 4.6: Biomarkers, including CRP and FC, are useful for adjunct assessment, and FC shows better correlation with endoscopic disease activity after surgery than CRP does (Evidence level 1a)	Voting agreement rate 46/47 (97.9%)

There is growing evidence for the use of FC in the postoperative monitoring of patients with CD [200], although this test is not yet indicated for postoperative monitoring in Japan. The correlation between FC and disease activity is consistent in patients who have and have not undergone surgery [217]. In the postoperative setting, FC is significantly correlated with endoscopic disease activity (*p* < 0.05) [201], and has a stronger correlation with disease activity than CRP does [217, 218].

Data from Japan have suggested that serial FC measurements can be used to identify patients who may have postoperative recurrence and would, therefore, benefit from ileocolonoscopy [219]. These authors suggested that the optimal FC threshold for postoperative patients was 140 μg/g [219].

However, this study included only 30 patients, and there is currently not enough evidence to recommend using FC rather than the strategy suggested by the POCER study. In addition, the optimal interval between FC measurements and the most appropriate threshold level of FC has yet to be fully defined [200], and may be affected by the choice of assay [220].

**Table:** 

Statement 4.7: In patients with symptoms of CD (pain and/or diarrhea) after surgical resection, other potential causes (such as gastrointestinal infection, bile acid malabsorption or bacterial overgrowth) should be ruled out and recurrent disease confirmed before making a decision on medical therapy (Evidence level 5)	Voting agreement rate 45/47 (95.7%)

In the early postoperative period, symptoms may indicate intra-abdominal sepsis, anastomotic leak or similar surgical complications [221, 222]. After this time, other potential causes of CD symptoms (particularly diarrhea) may include bile acid malabsorption (after ileocecal surgery), bacterial overgrowth, or enteric infections (e.g., associated with norovirus, *Clostridioides difficile*, or food-borne pathogens) [1, 221, 223]. In a patient with symptoms but no endoscopic evidence of active disease, other potential causes of gastrointestinal symptoms should be investigated and eliminated before intensifying treatment [1].

### Question 5. What are the considerations for de-escalation (i.e., discontinuing or reducing the dose of treatment) in patients with CD?

**Table:** 

Statement 5.1: De-escalation of anti-TNFα therapy is associated with an increased risk of relapse, which may be greater in patients receiving anti-TNFα monotherapy (Evidence level 3–4)	Voting agreement rate 46/48 (95.8%)

As summarized in Supplementary Table 17, relapse rates following discontinuation of anti-TNFα agents reported in clinical studies are high and increase over time. In these studies, a high proportion of patients generally received concomitant immunomodulator therapy (mostly azathioprine), whereas some patients were immunomodulator-naïve and, therefore, were receiving anti-TNFα monotherapy. In studies that examined the influence of concomitant immunomodulator therapy, some found only a small effect of concomitant immunomodulator therapy on the likelihood of relapse. However, a large retrospective, observational study by Casanova found concomitant immunomodulator therapy was significantly protective against relapse (HR 0.67; *p* = 0.003), which suggests patients who discontinue anti-TNFα monotherapy may be at greater risk of relapse [224].

In STORI, a pivotal, prospective, observational study of 115 patients with CD, relapse rates following discontinuation of infliximab were 44% at 1 year and 52% at 2 years [225]. Other observational studies of biologic therapies have reported 1-year relapse rates of 22–53% following discontinuation, with lower relapse rates typically seen in patients in remission [224, 226–231]. Furthermore, greater levels of remission in terms of duration and extent of mucosal healing and other endoscopic evidence of remission also appear to be protective against relapse following treatment de-escalation.

**Table:** 

5.2 Discontinuation of immunomodulator monotherapy is associated with an increased risk of relapse (Evidence level 2–4)	Voting agreement rate 45/48 (93.8%)

CD patients who discontinue immunomodulator monotherapy have been shown to have a high risk of relapse that increases over time (Supplementary Table 18) [232–238].

**Table:** 

Statement 5.3: Discontinuation of immunomodulator therapy may not increase the risk of relapse in patients receiving combination therapy with an anti-TNFα agent and immunomodulator who have achieved steroid-free clinical and biochemical remission for more than 6 months (Evidence level 2, 3, 5)	Voting agreement rate 44/47 (93.6%)

Concomitant use of immunomodulators with anti-TNFα therapy has been based on the principle of reduction in immunogenicity and improvement in efficacy [239]. Discontinuation of immunomodulator therapy in patients receiving combination treatment with anti-TNFα therapy should be considered separately to that of discontinuation of immunomodulator monotherapy. As shown in Supplementary Table 19 [240–243], results of different clinical studies are mixed but seem to suggest that immunomodulator discontinuation has less impact among patients on combination therapy than those receiving monotherapy. In particular, in the IMID study of 80 patients with CD, no long-term clinical benefit was observed from continuing immunomodulator therapy beyond 6 months in patients receiving scheduled infliximab, although patients who continued immunomodulator therapy had a lower frequency of infliximab antibodies and higher trough drug concentrations [243]. In support of these findings, a study in Japanese patients with CD also found that continuation of immunomodulator therapy for more than 6 months offered no clear benefit over scheduled anti-TNFα monotherapy in terms of corticosteroid-free clinical remission [240]. However, as with anti-TNFα therapy, the rate of relapse following immunomodulator cessation increases with longer periods of follow-up [244]. This has led to the suggestion that, in patients who receive combination therapy for more than 6 months, triple remission consisting of corticosteroid-free clinical remission, endoscopic remission, and serological remission allows consideration of thiopurine withdrawal [123]. Finally, the impact of immunomodulator discontinuation can be reduced by either partial withdrawal in terms of dose reduction rather than complete cessation or selective withdrawal in patients who have received concomitant treatment for longer periods. In any case, evidence from studies such as DIAMOND2 suggest that an individualized strategy of withdrawal should be implemented [123]. This should include ongoing assessment of measures of efficacy, including endoscopic or cross-sectional imaging parameters, biomarkers, and therapeutic drug monitoring.

**Table:** 

Statement 5.4: Key predictors of relapse after de-escalation include younger age, greater disease duration and severity, perianal disease, residual active lesion(s), elevated levels of CRP, FC, and leucocytes, and low hemoglobin levels. (Evidence level 2–4)	Voting agreement rate 45/47 (95.7%)

Predictors associated with increased relapse risk following discontinuation of anti-TNFα agents and immunomodulators are summarized and compared in Table 4.

**Table 4:** Predictors of relapse after de-escalation for patients receiving anti-TNFα and immunomodulator therapy

	Anti-TNFα therapy	Immunomodulator therapy
Frequently observed/apparent stronger association	Elevated CRP level [225, 245, 248, 249, 261, 262], elevated FC levels [225, 227, 245, 263], elevated leucocyte count [225, 227], low hemoglobin level [225, 245], younger age at diagnosis [245, 247, 248], perianal disease [133, 255, 256]	Elevated CRP level, elevated leukocyte or neutrophil count, low hemoglobin level [264]
Less frequently observed/possibly weaker association	Male gender [225], positive smoking status [245, 262], previous anti-TNFα therapy or dose escalation before discontinuation [263], presence of strictures or fistulas [265], higher trough levels (typically ≥ 2 µg/mL) [225, 245, 246]	High-risk disease with perianal involvement [244], younger age [232], male gender [232], short duration of remission [232], greater time without steroids [233], higher doses of azathioprine [237], thiopurine tapering before de-escalation [266], smoking cessation [235], lower or undetectable trough levels of anti-TNF agents in combination therapy [243, 244]

*CRP* C-reaction protein, *FC* fecal calprotectin, *TNF* tumor necrosis factor

Specific additional factors noted in various studies that appear to protect against relapse include: shorter disease duration [245, 246], mucosal healing [247], endoscopic and other evidence of remission [248, 249], optimization or continuation of thiopurine therapy [224, 250], and low or undetectable anti-TNFα levels at discontinuation [246, 247, 251, 252].

Based on these predictors of increased or decreased risk of relapse, patients in deep remission who have clinical, biomarker, and endoscopic factors associated with a lower risk of relapse have the greatest chance of favorable long-term prospects following anti-TNFα therapy discontinuation. However, relatively few patients in a real-world setting fulfil the criteria for withdrawal of biologic treatment even when they meet the lower risk criteria [253], and relatively few patients are able to achieve deep remission, which is the optimal state for considering treatment withdrawal [254].

Before discontinuing or reducing the intensity of any treatment, disease activity should be evaluated from a clinical perspective via a combined approach considering disease history, severity and extent to ensure appropriate patient selection. This approach includes an assessment of the likelihood of relapse based on the identified predictors. Selective patients may have acceptable outcomes with careful de-escalation, especially those who best fit the following profile:

- Older patients (generally > 22 to 25 years)
- Short disease duration or a short period between diagnosis and treatment
- Stable disease, lack of dose escalation or colonic surgery
- Evidence of remission, including endoscopic findings, mucosal healing and favorable biomarkers (e.g., low CRP, low FC).

In contrast, patients with perianal fistulas have been shown to have a high risk of relapse following cessation of anti-TNFα therapy and discontinuation is generally inadvisable in this specific population [133, 255, 256]. The presence or absence of a history of surgery and disease location have been considered as risk factors of relapse after discontinuation of anti-TNFα therapy but are not consistently reported in the literature. Therefore, more high-quality evidence is needed regarding these as potential risk factors for relapse.

**Table:** 

Statement 5.5: Patients who undergo treatment de-escalation should be monitored for relapse, especially in the initial period after discontinuation or reduction in dose (Evidence level 3, 5)	Voting agreement rate 46/47 (97.9%)

During treatment de-escalation, patients should be regularly monitored for relapse, especially in the initial period (6–12 months) after de-escalation during which the risk of relapse is greatest [244, 257]. Despite this, the duration during which the risk of relapse is greatest following discontinuation differs depending on prior treatment. Although the timing of peak relapse for different therapies is difficult to specify because of variation among study characteristics and findings, the risk of relapse appears to be high in the early period after discontinuation of monotherapy compared with combination therapy. There is a lack of consistent evidence to recommend a single optimal protocol for disease monitoring. However, a combined approach including monitoring of symptoms, biomarkers (e.g., CRP/FC), and/or endoscopy/imaging has been recommended by a recent European expert consensus panel [244]. A subanalysis of the STORI population found that elevated levels of CRP and FC were associated with an increased risk of short-term relapse following infliximab discontinuation [258]. Similarly, in a prospective cohort study of children with CD, FC levels > 250 μg/g accurately predicted clinical flares within 3 months [259]. As a consequence, expert consensus guidelines have suggested that CRP or FC should be used to monitor patients following discontinuation with elevated levels used as a trigger for further examination [244].

A systematic review and meta-analysis among CD patients in deep remission found that the rate of recapture of remission was relatively high (75.4%) after treatment was reinitiated [260].

## Conclusions

As T2T strategies become more prevalent in CD, it is important to identify clear indications for the escalation and de-escalation of treatment based on patient response. The current consensus document provides a framework and guidance for clinicians who are making these decisions in clinical practice and are specifically focused on making these decisions in different clinical scenarios, such as after surgery or in patients with perianal disease.

## Supplementary Information

Below is the link to the electronic supplementary material.

Supplementary file1 (DOCX 622 KB)
