# Supplementary material for: Treatment escalation and de-escalation decisions in Crohn’s disease: Delphi consensus recommendations from Japan, 2021
Source: J Gastroenterol. 2023 Feb 11;58(4):313–45. doi: 10.1007/s00535-023-01958-z (PMC10050046; doi:10.1007/s00535-023-01958-z)

# Supplementary Information

Treatment escalation and de-escalation decisions in Crohn’s disease: Delphi consensus recommendations from Japan, 2021

## Contents

Supplementary Table 1. TRADE consensus group (listed in alphabetic order)

Supplementary Table 2. Search strategies

Supplementary Table 3. Voting and consensus rates

Supplementary Table 4. Relationship between extraintestinal manifestations of CD and intestinal inflammation [50]. Adapted from Vavricka et al. 2015

Supplementary Table 5. Crohn’s Disease Activity Index [52]. Modified from Sandborn et al. 2002.

Supplementary Table 6. Harvey-Bradshaw Index [62].

Supplementary Table 7. SAR recommendations for when to use CTE and when to use MRE [83].

Supplementary Table 8. Accuracy of cross-sectional imaging techniques for grading CD severity [93].

Supplementary Table 9. Crohn’s disease Endoscopic Index of Severity (CDEIS) [95]. Modified from Mary et al. 1989.

Supplementary Table 10. Simplified Endoscopic Activity Score for Crohn’s disease (SES-CD) [96]. Modified from Daperno et al. 2004

Supplementary Table 11. CDEIS and SES-CD scores used to define disease severity [99]. Modified from Kucharski et al. 2016.

Supplementary Table 12. Cardiff Classification of Anal CD and 1992 Addition [145].

Supplementary Table 13. Perianal CD Activity Index (PDAI) [147].

Supplementary Table 14. Reported sensitivity and specificity of different cross-sectional imaging techniques for the diagnosis of stricturing CD [175, 179].

Supplementary Table 15. Definitions for patients at high risk of recurrence after resection

Supplementary Table 16. Modified Rutgeerts score [214].

Supplementary Table 17. Relapse rates associated with discontinuation of anti-TNFα therapy in clinical studies of CD

Supplementary Table 18. Relapse rates associated with discontinuation of azathioprine monotherapy in clinical studies of CD

Supplementary Table 19. Relapse findings associated with discontinuation of an immunomodulator from combination therapy with an anti-TNF α agent in clinical studies of CD

Supplementary Figure 1. Schema of simple and complex fistula anatomy (modified from Parks classification for consistency with AGA classification) [144].

## Supplementary Table 1. TRADE consensus group (listed in alphabetic order)

| **Member** | **Institution** |
| --- | --- |
| Akira Andoh | Division of Gastroenterology and Hematology, Department of Medicine, Shiga University of Medical Science, Shiga, Japan |
| Shigeki Bamba | Division of Digestive Endoscopy, Shiga University of Medical Science, Shiga, Japan |
| Motohiro Esaki* | Division of Gastroenterology, Department of Internal Medicine, Faculty of Medicine, Saga University, Saga, Japan |
| Mikihiro Fujiya | Division of Metabolism and Biosystemic Science, Gastroenterology and Hematology/Oncology, Department of Medicine, Asahikawa Medical University, Asahikawa, Hokkaido, Japan |
| Kitaro Futami | Department of Surgery, Matsunaga Hospital, Fukuoka, Japan |
| Keisuke Hata | Nihonbashi Muromachi Mitsui Tower Midtown Clinic, Tokyo, Japan |
| Fumihito Hirai* | Department of Gastroenterology and Medicine, Faculty of Medicine, Fukuoka University, Fukuoka, Japan |
| Sakiko Hiraoka | Department of Gastroenterology and Hepatology, Graduate School of Medicine, Dentistry and Pharmaceutical Sciences, Okayama University, Okayama, Japan |
| Tadakazu Hisamatsu* | Department of Gastroenterology and Hepatology, Kyorin University School of Medicine, Mitaka, Tokyo, Japan |
| Ryota Hokari | Department of Internal Medicine, National Defense Medical College​, Saitama, Japan |
| Shunji Ishihara | Department of Internal Medicine II, Shimane University Faculty of Medicine, Shimane, Japan |
| Soichiro Ishihara | Department of Surgical Oncology, Graduate School of Medicine, The University of Tokyo, Tokyo, Japan |
| Michio Itabashi | Department of Surgery, Division of Inflammatory Bowel Disease Surgery, Tokyo Women's Medical University, Tokyo, Japan |
| Yoichi Kakuta | Division of Gastroenterology, Tohoku University Graduate School of Medicine, Sendai, Japan |
| Jun Kato | Department of Gastroenterology, Graduate School of Medicine, Chiba University, Chiba, Japan |
| Shingo Kato | Department of Gastroenterology and Hepatology, Saitama Medical Center, Saitama Medical University, Saitama, Japan |
| Takehiko Katsurada | Department of Gastroenterology and Hepatology, Hokkaido University Graduate School of Medicine, Sapporo, Hokkaido, Japan |
| Kazuya Kitamura | Department of Gastroenterology, Kanazawa University Hospital, Kanazawa, Ishikawa, Japan |
| Kiyonori Kobayashi | Research and Development Center for New Medical Frontiers, Kitasato University School of Medicine, Kanagawa, Japan |
| Taku Kobayashi* | Center for Advanced IBD Research and Treatment, Kitasato University Kitasato Institute Hospital, Tokyo, Japan |
| Kazutaka Koganei | Department of Inflammatory Bowel Disease, Yokohama Municipal Citizen's Hospital, Kanagawa, Japan |
| Atsuo Maemoto | Inflammatory Bowel Disease Center, Sapporo Higashi Tokushukai Hospital, Sapporo, Hokkaido, Japan |
| Toshiyuki Matsui | Department of Gastroenterology, Coloproctology Center Takano Hospital, Kumamoto, Japan |
| Takayuki Matsumoto | Division of Gastroenterology and Hepatology, Department of Internal Medicine, School of Medicine, Iwate Medical University, Iwate, Japan |
| Katsuyoshi Matsuoka* | Division of Gastroenterology and Hepatology, Department of Internal Medicine, Toho University Sakura Medical Center, Sakura, Chiba, Japan |
| Minoru Matsuura* | Department of Gastroenterology and Hepatology, Kyorin University School of Medicine, Mitaka, Tokyo, Japan |
| Satoshi Motoya | Inflammatory Bowel Diseases Center, Sapporo-Kosei General Hospital, Sapporo, Hokkaido, Japan |
| Masakazu Nagahori | Department of Gastroenterology and Hepatology, Tokyo Medical and Dental University, Tokyo, Japan |
| Makoto Naganuma* | The Third Department of Internal Medicine, Division of Gastroenterology and Hepatology, Kansai Medical University, Osaka, Japan |
| Yuji Naito | Molecular Gastroenterology and Hepatology, Graduate School of Medical Science, Kyoto Prefectural University of Medicine, Kyoto, Japan |
| Shiro Nakamura | Second Department of Internal Medicine, Osaka Medical and Pharmaceutical University, Osaka, Japan |
| Hiroshi Nakase* | Department of Gastroenterology and Hepatology, Sapporo Medical University School of Medicine, Sapporo, Hokkaido, Japan |
| Haruhiko Ogata | Center for Diagnostic and Therapeutic Endoscopy, School of Medicine, Keio University, Tokyo, Japan |
| Kazuichi Okazaki | Kansai Medical University Kori Hospital, Osaka, Japan |
| Hirotake Sakuraba | Department of Gastroenterology and Hematology, Hirosaki University Graduate School of Medicine, Hirosaki, Aomori, Japan |
| Masayuki Saruta* | Division of Gastroenterology and Hepatology, Department of Internal Medicine, The Jikei University School of Medicine, Tokyo, Japan |
| Shinichiro Shinzaki | Department of Gastroenterology and Hepatology, Osaka University Graduate School of Medicine, Osaka, Japan |
| Ken Sugimoto | First Department of Medicine, Hamamatsu University School of Medicine, Hamamatsu, Shizuoka, Japan |
| Akira Sugita | Department of Inflammatory Bowel Disease, Yokohama Municipal Citizen's Hospital, Kanagawa, Japan |
| Yasuo Suzuki | Ginza Central Clinic, Tokyo, Japan |
| Kenichi Takahashi | Inflammatory Bowel Disease Center, Tohoku Rosai Hospital, Sendai, Miyagi, Japan |
| Tomohisa Takagi | Molecular Gastroenterology and Hepatology, Graduate School of Medical Science, Kyoto Prefectural University of Medicine, Kyoto, Japan |
| Kento Takenaka | Department of Gastroenterology and Hepatology, Tokyo Medical and Dental University, Tokyo, Japan |
| Ken Takeuchi | Department of Gastroenterology, IBD Center, Tsujinaka Hospital Kashiwanoha, Kashiwa, Japan |
| Kiichiro Tsuchiya* | Department of Gastroenterology, Faculty of Medicine, University of Tsukuba, Tsukuba, Japan |
| Tomoyuki Tsujikawa | Division of Gastroenterology, Kohka Public Hospital, Kohka, Japan |
| Motoi Uchino* | Division of Inflammatory Bowel Disease, Department of Gastroenterological Surgery, Hyogo College of Medicine, Nishinomiya, Hyogo, Japan |
| Fumiaki Ueno | Center for Gastroenterology and Inflammatory Bowel Disease, Ofuna Chuo Hospital, Kamakura, Kanagawa, Japan |
| Kenji Watanabe* | Center for Inflammatory Bowel Disease, Division of Internal Medicine, Hyogo College of Medicine, Nishinomiya, Hyogo, Japan |
| Mamoru Watanabe | Tokyo Medical and Dental University, Tokyo, Japan |
| Takayuki Yamamoto | Department of Surgery & Inflammatory Bowel Disease Center, Yokkaichi Hazu Medical Center, Yokkaichi, Mie, Japan |
| Kaoru Yokoyama | Department of Gastroenterology, Kitasato University School of Medicine, Sagamihara, Kanagawa, Japan |
| Atsushi Yoshida | Center for Gastroenterology and Inflammatory bowel disease, Ofuna Chuo Hospital, Kamakura, Kanagawa, Japan |
| Naoki Yoshimura | Ohori IBD Clinic, Tokyo, Japan |

TRADE, TReatment escAlation and DE-escalation decisions in Crohn’s disease

*Core panel member

## Supplementary Table 2. Search strategies

### 1. What are the criteria for treatment intensification in patients with luminal CD?

| # | Searches | Results | Notes |
| --- | --- | --- | --- |
| 1 | exp *Crohn Disease/ or ("Crohn* Diseas*" or "Crohn* Enterit*" or "Crohn* Enterocolit*" or "Crohn* Morbus" or "Morbus Crohn*" or "Cleron* Diseas*" or "Cleron* Enterit*" or "Cleron* Enterocolit*" or "Enterit* Regional*" or "Regional* Enterit*" or "Enterit* Granulomat*" or "Granulomat* Enterit*" or "Enterocolit* Regional*" or "Regional* Enterocolit*" or "Enterocolit* Granulomat*" or "Granulomat* Enterocolit*" or "Granulomat* Coliti*" or "Coliti* Granulomat*" or Ileocolit* or "Regional* Ileiti*" or "Ileiti* Regional*" or "Terminal* Ileiti*" or "Ileiti* Terminal*").mp. | 137173 | Line for Indication: Crohn’s disease |
| 2 | ("Penetrat* Crohn* Diseas*" or "Penetrat* Crohn* Enterit*" or "Penetrat* Crohn* Enterocolit*" or "Penetrat* Crohn* Morbus" or "Penetrat* Cleron* Diseas*" or "Penetrat* Cleron* Enterit*" or "Penetrat* Cleron* Enterocolit*" or "Penetrat* Enterit* Regional*" or "Penetrat* Regional* Enterit*" or "Penetrat* Enterit* Granulomat*" or "Penetrat* Granulomat* Enterit*" or "Penetrat* Enterocolit* Regional*" or "Penetrat* Regional* Enterocolit*" or "Penetrat* Enterocolit* Granulomat*" or "Penetrat* Granulomat* Enterocolit*" or "Penetrat* Granulomat* Coliti*" or "Penetrat* Coliti* Granulomat*" or "Penetrat* Ileocolit*" or "Penetrat* Regional* Ileiti*" or "Penetrat* Ileiti* Regional*" or "Penetrat* Terminal* Ileiti*" or "Penetrat* Ileiti* Terminal*" or "Penetrat* CD" or "Fibrostenos* Crohn* Diseas*" or "Fibrostenos* Crohn* Enterit*" or "Fibrostenos* Crohn* Enterocolit*" or "Fibrostenos* Crohn* Morbus" or "Fibrostenos* Cleron* Diseas*" or "Fibrostenos* Cleron* Enterit*" or "Fibrostenos* Cleron* Enterocolit*" or "Fibrostenos* Enterit* Regional*" or "Fibrostenos* Regional* Enterit*" or "Fibrostenos* Enterit* Granulomat*" or "Fibrostenos* Granulomat* Enterit*" or "Fibrostenos* Enterocolit* Regional*" or "Fibrostenos* Regional* Enterocolit*" or "Fibrostenos* Enterocolit* Granulomat*" or "Fibrostenos* Granulomat* Enterocolit*" or "Fibrostenos* Granulomat* Coliti*" or "Fibrostenos* Coliti* Granulomat*" or "Fibrostenos* Ileocolit*" or "Fibrostenos* Regional* Ileiti*" or "Fibrostenos* Ileiti* Regional*" or "Fibrostenos* Terminal* Ileiti*" or "Fibrostenos* Ileiti* Terminal*" or "Fibrostenos* CD").mp. | 280 | Free-text terms for:  - Penetrating Crohn’s Disease.  - Fibrostenosing Crohn’s Disease |
| 3 | (("Crohn* Diseas*" or "Crohn* Enterit*" or "Crohn* Enterocolit*" or "Crohn* Morbus" or "Morbus Crohn*" or "Cleron* Diseas*" or "Cleron* Enterit*" or "Cleron* Enterocolit*" or "Enterit* Regional*" or "Regional* Enterit*" or "Enterit* Granulomat*" or "Granulomat* Enterit*" or "Enterocolit* Regional*" or "Regional* Enterocolit*" or "Enterocolit* Granulomat*" or "Granulomat* Enterocolit*" or "Granulomat* Coliti*" or "Coliti* Granulomat*" or Ileocolit* or "Regional* Ileiti*" or "Ileiti* Regional*" or "Terminal* Ileiti*" or "Ileiti* Terminal*") adj3 (Penetrat* or Fibrostenos*)).mp. | 212 | Free-text terms for:  - Penetrating Crohn’s Disease.  - Fibrostenosing Crohn’s Disease |
| 4 | 1 NOT (2 OR 3) | 136816 | [Crohn's Disease] Excluding/NOT [Penetrating and Fibrostenosing Crohn’s Disease]  MAIN SET |
| 5 | ((Intensi* or Escalat* or Boost* or Switch* or Added or Adding or Addition* or Supplemen* or Strength* or Amplif*) adj3 (Treat* or Therap* or Medic* or Drug* or Intervent*)).mp. | 53681 | Free-text terms for:  -Intensified Treatment.  -Intensified Therapy. |
| 6 | ("Intensi* Treat*" or "Treat* Intensi*" or "Intensi* Therap*" or "Therap* Intensi*" or "Intensi* Medic*" or "Medic* Intensi*" or "Intensi* Drug*" or "Drug* Intensi*" or "Intensi* Intervent*" or "Intervent* Intensi*" or "Escalat* Treat*" or "Treat* Escalat*" or "Escalat* Therap*" or "Therap* Escalat*" or "Escalat* Medic*" or "Medic* Escalat*" or "Escalat* Drug*" or "Drug* Escalat*" or "Escalat* Intervent*" or "Intervent* Escalat*" or "Switch* Treat*" or "Treat* Switch*" or "Switch* Therap*" or "Therap* Switch*" or "Switch* Medic*" or "Medic* Switch*" or "Switch* Drug*" or "Drug* Switch*" or "Switch* Intervent*" or "Intervent* Switch*" or "Amplif* Treat*" or "Treat* Amplif*" or "Amplif* Therap*" or "Therap* Amplif*" or "Boost* Treat*" or "Treat* Boost*" or "Boost* Therap*" or "Therap* Boost*" or "Added Treat*" or "Treat* Added" or "Added Therap*" or "Therap* Added" or "Adding Treat*" or "Adding Therap*" or "Addition* Treat*" or "Treat* Addition*" or "Addition* Therap*" or "Therap* Addition*" or "Addition* Medic*" or "Medic* Addition*" or "Supplemen* Treat*" or "Treat* Supplemen*" or "Supplemen* Therap*" or "Therap* Supplemen*" or "Supplemen* Medic*" or "Medic* Supplemen*" or "Supplemen* Drug*" or "Drug* Supplemen*" or "Increas* Treat*" or "Treat* Increas*" or "Increas* Therap*" or "Therap* Increas*" or "Increas* Medic*" or "Medic* Increas*" or "Strength* Treat*" or "Treat* Strength*" or "Strength* Therap*" or "Therap* Strength*" or "Strength* Medic*" or "Medic* Strength*").mp. | 211029 | Free-text terms for:  -Intensified Treatment.  -Intensified Therapy. |
| 7 | 4 AND (5 OR 6) | 4117 | Main set + treatment intensificaiton |
| 8 | Limit 7 to English language | 3906 | Set limiting to English |
| 9 | Limit 8 to yr=”2014-Current” | 2315 | Publications from 2014 |
| 10 | (animal* or rat or rats or mouse or mice or murine* or xenograft* or "guinea pig*" or guinea-pig* or guineapig* or rabbit* or monkey* or dog or dogs or canine* or cat or cats or feline* or pig or pigs or sheep or "in vitro*" or in-vitro* or invitro* or "cell* culture*" or cell-culture* or cell?culture* or "cell* line*" or cell-line* or cell?line* or culture*).ti,ab,sh. | 14031269 |  |
| 11 | 9 NOT 10 | 2120 | Set limiting to humans |
| 12 | Remove duplicates from 11 | 1727 | Luminal Crohn’s set |

### 2. What are the criteria for treatment intensification in patients with CD with perianal or fistulizing disease?

Originally, we planned to have two separate questions - one on perianal disease and one on fistulizing disease, but the content contained considerable overlap, so the two topics were combined. The original search strategies are as below:

| # | Searches | Results | Notes |
| --- | --- | --- | --- |
| Perianal disease | | | |
| 1 | exp *Crohn Disease/ or ("Crohn* Diseas*" or "Crohn* Enterit*" or "Crohn* Enterocolit*" or "Crohn* Morbus" or "Morbus Crohn*" or "Cleron* Diseas*" or "Cleron* Enterit*" or "Cleron* Enterocolit*" or "Enterit* Regional*" or "Regional* Enterit*" or "Enterit* Granulomat*" or "Granulomat* Enterit*" or "Enterocolit* Regional*" or "Regional* Enterocolit*" or "Enterocolit* Granulomat*" or "Granulomat* Enterocolit*" or "Granulomat* Coliti*" or "Coliti* Granulomat*" or Ileocolit* or "Regional* Ileiti*" or "Ileiti* Regional*" or "Terminal* Ileiti*" or "Ileiti* Terminal*").mp. | 138408 | Line for Indication: Crohn’s disease |
| 2 | ((anus* or anal or anally or perianal* or anorectal*) adj3 (lesion* or ulcer* or sore* or abscess*)).mp. | 8073 | Free-text terms for:  - Anal lesions |
| 3 | ("anus* lesion*" or "anal lesion*" or "perianal* lesion*" or "anorectal* lesion*" or "anus* ulcer*" or "anal ulcer*" or "perianal* ulcer*" or "anorectal* ulcer*" or "anus* sore*" or "anal sore*" or "perianal* sore*" or "anorectal* sore*" or "anus* abscess*" or "anal abscess*" or "perianal* abscess*" or "anorectal* abscess*").mp. | 5234 | Free-text terms for:  - Anal lesions |
| 4 | 1 AND (2 OR 3) | 1892 | Crohn’s disease and Anal lesions  MAIN SET |
| 5 | ((Intensi* or Escalat* or Boost* or Switch* or Added or Adding or Addition* or Supplemen* or Strength* or Amplif*) adj3 (Treat* or Therap* or Medic* or Drug* or Intervent*)).mp. | 652030 | Free-text terms for:  -Intensified Treatment.  -Intensified Therapy. |
| 6 | ("Intensi* Treat*" or "Treat* Intensi*" or "Intensi* Therap*" or "Therap* Intensi*" or "Intensi* Medic*" or "Medic* Intensi*" or "Intensi* Drug*" or "Drug* Intensi*" or "Intensi* Intervent*" or "Intervent* Intensi*" or "Escalat* Treat*" or "Treat* Escalat*" or "Escalat* Therap*" or "Therap* Escalat*" or "Escalat* Medic*" or "Medic* Escalat*" or "Escalat* Drug*" or "Drug* Escalat*" or "Escalat* Intervent*" or "Intervent* Escalat*" or "Switch* Treat*" or "Treat* Switch*" or "Switch* Therap*" or "Therap* Switch*" or "Switch* Medic*" or "Medic* Switch*" or "Switch* Drug*" or "Drug* Switch*" or "Switch* Intervent*" or "Intervent* Switch*" or "Amplif* Treat*" or "Treat* Amplif*" or "Amplif* Therap*" or "Therap* Amplif*" or "Boost* Treat*" or "Treat* Boost*" or "Boost* Therap*" or "Therap* Boost*" or "Added Treat*" or "Treat* Added" or "Added Therap*" or "Therap* Added" or "Adding Treat*" or "Adding Therap*" or "Addition* Treat*" or "Treat* Addition*" or "Addition* Therap*" or "Therap* Addition*" or "Addition* Medic*" or "Medic* Addition*" or "Supplemen* Treat*" or "Treat* Supplemen*" or "Supplemen* Therap*" or "Therap* Supplemen*" or "Supplemen* Medic*" or "Medic* Supplemen*" or "Supplemen* Drug*" or "Drug* Supplemen*" or "Increas* Treat*" or "Treat* Increas*" or "Increas* Therap*" or "Therap* Increas*" or "Increas* Medic*" or "Medic* Increas*" or "Strength* Treat*" or "Treat* Strength*" or "Strength* Therap*" or "Therap* Strength*" or "Strength* Medic*" or "Medic* Strength*").mp. | 306364 | Free-text terms for:  -Intensified Treatment.  -Intensified Therapy. |
| 7 | 4 AND (5 OR 6) | 95 | Main set + treatment intensification |
| 8 | Limit 7 to English language | 92 | Set limiting to English |
| 9 | Limit 8 to yr=”1998-Current” | 2315 | Publications from 1998 |
| 10 | (animal* or rat or rats or mouse or mice or murine* or xenograft* or "guinea pig*" or guinea-pig* or guineapig* or rabbit* or monkey* or dog or dogs or canine* or cat or cats or feline* or pig or pigs or sheep or "in vitro*" or in-vitro* or invitro* or "cell* culture*" or cell-culture* or cell?culture* or "cell* line*" or cell-line* or cell?line* or culture*).ti,ab,sh. | 14129465 |  |
| 11 | 9 NOT 10 | 88 | Set limiting to humans |
| 12 | Remove duplicates from 11 | 78 | Perianal Crohn’s set |
| Fistulizing disease | | | |
| 1 | exp *Crohn Disease/ or ("Crohn* Diseas*" or "Crohn* Enterit*" or "Crohn* Enterocolit*" or "Crohn* Morbus" or "Morbus Crohn*" or "Cleron* Diseas*" or "Cleron* Enterit*" or "Cleron* Enterocolit*" or "Enterit* Regional*" or "Regional* Enterit*" or "Enterit* Granulomat*" or "Granulomat* Enterit*" or "Enterocolit* Regional*" or "Regional* Enterocolit*" or "Enterocolit* Granulomat*" or "Granulomat* Enterocolit*" or "Granulomat* Coliti*" or "Coliti* Granulomat*" or Ileocolit* or "Regional* Ileiti*" or "Ileiti* Regional*" or "Terminal* Ileiti*" or "Ileiti* Terminal*").mp. | 138408 | Line for Indication: Crohn’s disease |
| 2 | exp *Fistula/ or Fistul*.mp. | 239415 | Free-text terms for:  - Fistulas |
| 3 | 1 AND 2 | 12080 | Crohn’s disease and fistula  MAIN SET |
| 4 | ((Intensi* or Escalat* or Boost* or Switch* or Added or Adding or Addition* or Supplemen* or Strength* or Amplif*) adj3 (Treat* or Therap* or Medic* or Drug* or Intervent*)).mp. | 652030 | Free-text terms for:  -Intensified Treatment.  -Intensified Therapy. |
| 5 | ("Intensi* Treat*" or "Treat* Intensi*" or "Intensi* Therap*" or "Therap* Intensi*" or "Intensi* Medic*" or "Medic* Intensi*" or "Intensi* Drug*" or "Drug* Intensi*" or "Intensi* Intervent*" or "Intervent* Intensi*" or "Escalat* Treat*" or "Treat* Escalat*" or "Escalat* Therap*" or "Therap* Escalat*" or "Escalat* Medic*" or "Medic* Escalat*" or "Escalat* Drug*" or "Drug* Escalat*" or "Escalat* Intervent*" or "Intervent* Escalat*" or "Switch* Treat*" or "Treat* Switch*" or "Switch* Therap*" or "Therap* Switch*" or "Switch* Medic*" or "Medic* Switch*" or "Switch* Drug*" or "Drug* Switch*" or "Switch* Intervent*" or "Intervent* Switch*" or "Amplif* Treat*" or "Treat* Amplif*" or "Amplif* Therap*" or "Therap* Amplif*" or "Boost* Treat*" or "Treat* Boost*" or "Boost* Therap*" or "Therap* Boost*" or "Added Treat*" or "Treat* Added" or "Added Therap*" or "Therap* Added" or "Adding Treat*" or "Adding Therap*" or "Addition* Treat*" or "Treat* Addition*" or "Addition* Therap*" or "Therap* Addition*" or "Addition* Medic*" or "Medic* Addition*" or "Supplemen* Treat*" or "Treat* Supplemen*" or "Supplemen* Therap*" or "Therap* Supplemen*" or "Supplemen* Medic*" or "Medic* Supplemen*" or "Supplemen* Drug*" or "Drug* Supplemen*" or "Increas* Treat*" or "Treat* Increas*" or "Increas* Therap*" or "Therap* Increas*" or "Increas* Medic*" or "Medic* Increas*" or "Strength* Treat*" or "Treat* Strength*" or "Strength* Therap*" or "Therap* Strength*" or "Strength* Medic*" or "Medic* Strength*").mp. | 306364 | Free-text terms for:  -Intensified Treatment.  -Intensified Therapy. |
| 6 | 3 AND (4 OR 5) | 539 | Main set + treatment intensification |
| 7 | Limit 6 to English language | 498 | Set limiting to English |
| 8 | Limit 7 to yr=”1998-Current” | 487 | Publications from 1998 |
| 9 | (animal* or rat or rats or mouse or mice or murine* or xenograft* or "guinea pig*" or guinea-pig* or guineapig* or rabbit* or monkey* or dog or dogs or canine* or cat or cats or feline* or pig or pigs or sheep or "in vitro*" or in-vitro* or invitro* or "cell* culture*" or cell-culture* or cell?culture* or "cell* line*" or cell-line* or cell?line* or culture*).ti,ab,sh. | 14129465 |  |
| 10 | 8 NOT 9 | 468 | Set limiting to humans |
| 11 | Remove duplicates from 10 | 375 | Fistulizing Crohn’s set |

### 3. What are the criteria for treatment intensification in patients with CD with small bowel stenosis?

| # | Searches | Results | Notes |
| --- | --- | --- | --- |
| 1 | exp *Crohn Disease/ or ("Crohn* Diseas*" or "Crohn* Enterit*" or "Crohn* Enterocolit*" or "Crohn* Morbus" or "Morbus Crohn*" or "Cleron* Diseas*" or "Cleron* Enterit*" or "Cleron* Enterocolit*" or "Enterit* Regional*" or "Regional* Enterit*" or "Enterit* Granulomat*" or "Granulomat* Enterit*" or "Enterocolit* Regional*" or "Regional* Enterocolit*" or "Enterocolit* Granulomat*" or "Granulomat* Enterocolit*" or "Granulomat* Coliti*" or "Coliti* Granulomat*" or Ileocolit* or "Regional* Ileiti*" or "Ileiti* Regional*" or "Terminal* Ileiti*" or "Ileiti* Terminal*").mp. | 138408 | Line for Indication: Crohn’s disease |
| 2 | (("small intestin*" or "small bowel*" or duoden* or jejun* or ile*) adj3 (stenos?s* or arctation* or coarctation* or stricture* or "pathol* constrict*")).mp. | 6051 | Free-text terms for:  - Small bowel stenosis |
| 3 | ("small intestin* stenos?s*" or "small intestin* arctation*" or "small intestin* coarctation*" or "small intestin* stricture*" or "small intestin* pathol* constrict*" or "small intestin* constrict*" or "small bowel* stenos?s*" or "small bowel* arctation*" or "small bowel* coarctation*" or "small bowel* stricture*" or "small bowel* pathol* constrict*" or "small bowel* constrict*" or "duoden* stenos?s*" or "duoden* arctation*" or "duoden* coarctation*" or "duoden* stricture*" or "duoden* pathol* constrict*" or "duoden* constrict*" or "jejun* stenos?s*" or "jejun* arctation*" or "jejun* coarctation*" or "jejun* stricture*" or "jejun* pathol* constrict*" or "jejun* constrict*" or "ile* stenos?s*" or "ile* arctation*" or "ile* coarctation*" or "ile* stricture*" or "ile* pathol* constrict*" or "ile* constrict*").mp. | 3767 | Free-text terms for:  - Small bowel stenosis |
| 4 | 1 NOT (2 OR 3) | 1447 | [Crohn's Disease] and [Small bowel stenosis]  MAIN SET |
| 5 | ((Intensi* or Escalat* or Boost* or Switch* or Added or Adding or Addition* or Supplemen* or Strength* or Amplif*) adj3 (Treat* or Therap* or Medic* or Drug* or Intervent*)).mp. | 652030 | Free-text terms for:  -Intensified Treatment.  -Intensified Therapy. |
| 6 | ("Intensi* Treat*" or "Treat* Intensi*" or "Intensi* Therap*" or "Therap* Intensi*" or "Intensi* Medic*" or "Medic* Intensi*" or "Intensi* Drug*" or "Drug* Intensi*" or "Intensi* Intervent*" or "Intervent* Intensi*" or "Escalat* Treat*" or "Treat* Escalat*" or "Escalat* Therap*" or "Therap* Escalat*" or "Escalat* Medic*" or "Medic* Escalat*" or "Escalat* Drug*" or "Drug* Escalat*" or "Escalat* Intervent*" or "Intervent* Escalat*" or "Switch* Treat*" or "Treat* Switch*" or "Switch* Therap*" or "Therap* Switch*" or "Switch* Medic*" or "Medic* Switch*" or "Switch* Drug*" or "Drug* Switch*" or "Switch* Intervent*" or "Intervent* Switch*" or "Amplif* Treat*" or "Treat* Amplif*" or "Amplif* Therap*" or "Therap* Amplif*" or "Boost* Treat*" or "Treat* Boost*" or "Boost* Therap*" or "Therap* Boost*" or "Added Treat*" or "Treat* Added" or "Added Therap*" or "Therap* Added" or "Adding Treat*" or "Adding Therap*" or "Addition* Treat*" or "Treat* Addition*" or "Addition* Therap*" or "Therap* Addition*" or "Addition* Medic*" or "Medic* Addition*" or "Supplemen* Treat*" or "Treat* Supplemen*" or "Supplemen* Therap*" or "Therap* Supplemen*" or "Supplemen* Medic*" or "Medic* Supplemen*" or "Supplemen* Drug*" or "Drug* Supplemen*" or "Increas* Treat*" or "Treat* Increas*" or "Increas* Therap*" or "Therap* Increas*" or "Increas* Medic*" or "Medic* Increas*" or "Strength* Treat*" or "Treat* Strength*" or "Strength* Therap*" or "Therap* Strength*" or "Strength* Medic*" or "Medic* Strength*").mp. | 306364 | Free-text terms for:  -Intensified Treatment.  -Intensified Therapy. |
| 7 | 4 AND (5 OR 6) | 40 | Main set + treatment intensificaiton |
| 8 | Limit 7 to English language | 39 | Set limiting to English |
| 9 | Limit 8 to yr=”1998-Current” | 39 | Publications from 1998 |
| 10 | (animal* or rat or rats or mouse or mice or murine* or xenograft* or "guinea pig*" or guinea-pig* or guineapig* or rabbit* or monkey* or dog or dogs or canine* or cat or cats or feline* or pig or pigs or sheep or "in vitro*" or in-vitro* or invitro* or "cell* culture*" or cell-culture* or cell?culture* or "cell* line*" or cell-line* or cell?line* or culture*).ti,ab,sh. | 14129465 |  |
| 11 | 9 NOT 10 | 38 | Set limiting to humans |
| 12 | Remove duplicates from 11 | 34 | Small bowel stenosis set |

### 4. What are the criteria for treatment intensification in patients with CD in the postoperative setting?

| # | Searches | Results | Notes |
| --- | --- | --- | --- |
| 1 | exp *Crohn Disease/ or ("Crohn* Diseas*" or "Crohn* Enterit*" or "Crohn* Enterocolit*" or "Crohn* Morbus" or "Morbus Crohn*" or "Cleron* Diseas*" or "Cleron* Enterit*" or "Cleron* Enterocolit*" or "Enterit* Regional*" or "Regional* Enterit*" or "Enterit* Granulomat*" or "Granulomat* Enterit*" or "Enterocolit* Regional*" or "Regional* Enterocolit*" or "Enterocolit* Granulomat*" or "Granulomat* Enterocolit*" or "Granulomat* Coliti*" or "Coliti* Granulomat*" or Ileocolit* or "Regional* Ileiti*" or "Ileiti* Regional*" or "Terminal* Ileiti*" or "Ileiti* Terminal*").mp. | 138408 | Line for Indication: Crohn’s disease |
| 2 | ((Post* or After* or Follow*) adj3 (Operat* or Surg* or Resect*)).mp. | 1293248 | Free-text terms for:  - Postoperative |
| 3 | ("Post Operat*" or Post-operat* or Postoperat* or "After Operat*" or After-operat* or "Follow* Operat*" or "Post Surg*" or Post-surg* or Postsurg* or "After Surg*" or After-surg* or "Follow Surg*" or "Post Resect*" or Post-resect* or Postresect* or "After Resect*" or After-resect* or "Follow* Resect*").mp. | 2095139 | Free-text terms for:  - Postoperative |
| 4 | 1 NOT (2 OR 3) | 12069 | [Crohn's Disease] and [postoperative]  MAIN SET |
| 5 | ((Intensi* or Escalat* or Boost* or Switch* or Added or Adding or Addition* or Supplemen* or Strength* or Amplif*) adj3 (Treat* or Therap* or Medic* or Drug* or Intervent*)).mp. | 652030 | Free-text terms for:  -Intensified Treatment.  -Intensified Therapy. |
| 6 | ("Intensi* Treat*" or "Treat* Intensi*" or "Intensi* Therap*" or "Therap* Intensi*" or "Intensi* Medic*" or "Medic* Intensi*" or "Intensi* Drug*" or "Drug* Intensi*" or "Intensi* Intervent*" or "Intervent* Intensi*" or "Escalat* Treat*" or "Treat* Escalat*" or "Escalat* Therap*" or "Therap* Escalat*" or "Escalat* Medic*" or "Medic* Escalat*" or "Escalat* Drug*" or "Drug* Escalat*" or "Escalat* Intervent*" or "Intervent* Escalat*" or "Switch* Treat*" or "Treat* Switch*" or "Switch* Therap*" or "Therap* Switch*" or "Switch* Medic*" or "Medic* Switch*" or "Switch* Drug*" or "Drug* Switch*" or "Switch* Intervent*" or "Intervent* Switch*" or "Amplif* Treat*" or "Treat* Amplif*" or "Amplif* Therap*" or "Therap* Amplif*" or "Boost* Treat*" or "Treat* Boost*" or "Boost* Therap*" or "Therap* Boost*" or "Added Treat*" or "Treat* Added" or "Added Therap*" or "Therap* Added" or "Adding Treat*" or "Adding Therap*" or "Addition* Treat*" or "Treat* Addition*" or "Addition* Therap*" or "Therap* Addition*" or "Addition* Medic*" or "Medic* Addition*" or "Supplemen* Treat*" or "Treat* Supplemen*" or "Supplemen* Therap*" or "Therap* Supplemen*" or "Supplemen* Medic*" or "Medic* Supplemen*" or "Supplemen* Drug*" or "Drug* Supplemen*" or "Increas* Treat*" or "Treat* Increas*" or "Increas* Therap*" or "Therap* Increas*" or "Increas* Medic*" or "Medic* Increas*" or "Strength* Treat*" or "Treat* Strength*" or "Strength* Therap*" or "Therap* Strength*" or "Strength* Medic*" or "Medic* Strength*").mp. | 306364 | Free-text terms for:  -Intensified Treatment.  -Intensified Therapy. |
| 7 | 4 AND (5 OR 6) | 406 | Main set + treatment intensificaiton |
| 8 | Limit 7 to English language | 382 | Set limiting to English |
| 9 | Limit 8 to yr=”1998-Current” | 369 | Publications from 1998 |
| 10 | (animal* or rat or rats or mouse or mice or murine* or xenograft* or "guinea pig*" or guinea-pig* or guineapig* or rabbit* or monkey* or dog or dogs or canine* or cat or cats or feline* or pig or pigs or sheep or "in vitro*" or in-vitro* or invitro* or "cell* culture*" or cell-culture* or cell?culture* or "cell* line*" or cell-line* or cell?line* or culture*).ti,ab,sh. | 14129465 |  |
| 11 | 9 NOT 10 | 360 | Set limiting to humans |
| 12 | Remove duplicates from 11 | 285 | Small bowel stenosis set |

### 5. What are the criteria for discontinuing or reducing the dose of treatment (i.e., de-escalation) in patients with CD?

| # | Searches | Results | Notes |
| --- | --- | --- | --- |
| 1 | exp *Crohn Disease/ or ("Crohn* Diseas*" or "Crohn* Enterit*" or "Crohn* Enterocolit*" or "Crohn* Morbus" or "Morbus Crohn*" or "Cleron* Diseas*" or "Cleron* Enterit*" or "Cleron* Enterocolit*" or "Enterit* Regional*" or "Regional* Enterit*" or "Enterit* Granulomat*" or "Granulomat* Enterit*" or "Enterocolit* Regional*" or "Regional* Enterocolit*" or "Enterocolit* Granulomat*" or "Granulomat* Enterocolit*" or "Granulomat* Coliti*" or "Coliti* Granulomat*" or Ileocolit* or "Regional* Ileiti*" or "Ileiti* Regional*" or "Terminal* Ileiti*" or "Ileiti* Terminal*").mp. | 137173 | Line for Indication: Crohn’s disease |
| 2 | (((Stop* or Discont* or Withdraw* or Reduc* or Decreas*) adj2 (Treat* or Therap* or Dose* or Dosage* or Dosing* or "Dose* frequenc*" or "Dosing* frequenc*" or Drug* or admin* or "admin* frequenc*")) or (Change* adj2 ("Dose* frequenc*" or Dosage* or "Dosing* frequenc*" or admin* or "admin* frequenc*"))).ti,ab,kw. | 544959 | Free-text terms for:  - Stopping treatment.  - Reducing dose |
| 3 | ("Stop* Treat*" or "Stop* Therap*" or "Stop Dose*" or "Stop* Dosage*" or "Stop* Dosing*" or "Stop* Drug*" or "Stop* Drug* Dose*" or "Stop* Drug* Dosage*" or "Stop* Drug* Dosing*" or "Stop* Drug* admin*" or "Stop* Drug* admin* frequenc*" or "Stop* admin* frequenc*" or "Stop* admin*" or "Discont* Treat*" or "Discont* Therap*" or "Discont* Dose*" or "Discont* Dosage*" or "Discont* Dosing*" or "Discont* Drug*" or "Discont* Drug* Dose*" or "Discont* Drug* Dosage*" or "Discont* Drug* Dosing*" or "Discont* Drug* admin*" or "Discont* Drug* admin* frequenc*" or "Discont* admin* frequenc*" or "Discont* admin*" or "Withdraw* Treat*" or "Withdraw* Therap*" or "Withdraw* Drug*" or "Reduc* Treat*" or "Reduc* Therap*" or "Reduc* Dose*" or "Reduc* Dosage*" or "Reduc* Dosing*" or "Reduc* Drug*" or "Reduc* Drug* Dose*" or "Reduc* Drug* Dosage*" or "Reduc* Drug* Dosing*" or "Reduc* Drug* admin*" or "Reduc* Drug* admin* frequenc*" or "Reduc* admin* frequenc*" or "Reduc* admin*" or "Decreas* Treat*" or "Decreas* Therap*" or "Decreas* Dose*" or "Decreas* Dosage*" or "Decreas* Dosing*" or "Decrease Drug*" or "Decreas* Drug* Dose*" or "Decreas* Drug* Dosage*" or "Decreas* Drug* Dosing*" or "Decreas* Drug* admin*" or "Decreas* Drug* admin* frequenc*" or "Decreas* admin* frequenc*" or "Decreas* admin*").ti,ab,kw. | 71729 | Free-text terms for:  - Stopping treatment.  - Reducing dose |
| 4 | 1 AND (2 OR 3) | 3524 | [Crohn's Disease] Excluding/NOT [De-escalating treatment]  MAIN SET |
| 5 | Limit 4 to English language | 3355 | Set limiting to English |
| 6 | Limit 5 to yr=”2014-Current” | 1811 | Publications from 2014 |
| 7 | (animal* or rat or rats or mouse or mice or murine* or xenograft* or "guinea pig*" or guinea-pig* or guineapig* or rabbit* or monkey* or dog or dogs or canine* or cat or cats or feline* or pig or pigs or sheep or "in vitro*" or in-vitro* or invitro* or "cell* culture*" or cell-culture* or cell?culture* or "cell* line*" or cell-line* or cell?line* or culture*).ti,ab,sh. | 14031269 |  |
| 8 | 6 NOT 7 | 1602 | Set limiting to humans |
| 9 | Remove duplicates from 11 | 1298 | Treatment de-escalation |

## Supplementary Table 3. Voting and consensus rates

| **Statement** | **Consensus rate** | **Voting rate** |
| --- | --- | --- |
| 1.1 | 89.1% (41/46) | 100% (46/46) |
| 1.2 | 95.5% (42/44) | 97.8% (44/45) |
| 1.3 | 95.5% (42/44) | 97.8% (44/45) |
| 1.4 | 100% (44/44) | 97.8% (44/45) |
| 1.5 | 100% (45/45) | 95.7% (45/47) |
| 1.6 | 97.9% (46/47) | 100% (47/47) |
| 1.7 | 100% (47/47) | 100% (47/47) |
| 1.8 | 100% (47/47) | 100% (47/47) |
| 1.9 | 100% (47/47) | 100% (47/47) |
| 1.10 | 97.9% (46/47) | 100% (47/47) |
| 1.11 | 97.9% (46/47) | 100% (47/47) |
| 1.12 | 82.2% (37/45) | 95.7% (45/47) |
| 1.13 | 100% (45/45) | 95.7% (45/47) |
| 1.14 | 100% (45/45) | 95.7% (45/47) |
| 1.15 | 100% (46/46) | 97.9% (46/47) |
| 1.16 | 97.8% (45/46) | 97.9% (46/47) |
| 1.17 | 100.0% (41/41) | 97.6% (41/42) |
| 2.1 | 100% (45/45) | 95.7% (45/47) |
| 2.2 | 97.9% (46/47) | 100% (47/47) |
| 2.3 | 100% (46/46) | 100% (46/46) |
| 2.4 | 100% (46/46) | 100% (46/46) |
| 2.5 | 100% (45/45) | 97.8% (45/46) |
| 2.6 | 97.3% (36/37) | 100.0% (37/37) |
| 3.1 | 97.8% (45/46) | 97.9% (46/47) |
| 3.2 | 95.5% (42/44) | 93.6% (44/47) |
| 3.3 | 97.8% (45/46) | 97.9% (46/47) |
| 3.4 | 84.8% (39/46) | 97.9% (46/47) |
| 3.5 | 100% (46/46) | 97.9% (46/47) |
| 3.6 | 95.6% (43/45) | 95.7% (45/47) |
| 4.1 | 97.9% (46/47) | 97.9% (47/48) |
| 4.2 | 95.8% (46/48) | 100% (48/48) |
| 4.3 | 100% (47/47) | 97.9% (47/48) |
| 4.4 | 93.5% (43/46) | 95.8% (46/48) |
| 4.5 | 100% (47/47) | 97.9% (47/48) |
| 4.6 | 97.9% (46/47) | 97.9% (47/48) |
| 4.7 | 95.7% (45/47) | 97.9% (47/48) |
| 5.1 | 95.8% (46/48) | 100% (48/48) |
| 5.2 | 93.8% (45/48) | 100% (48/48) |
| 5.3 | 93.6% (44/47) | 97.9% (47/48) |
| 5.4 | 95.7% (45/47) | 97.9% (47/48) |
| 5.5 | 97.9% (46/47) | 97.9% (47/48) |

## Supplementary Table 4. Relationship between extraintestinal manifestations of CD and intestinal inflammation [50]. Adapted from Vavricka et al. 2015

| **Occurs in parallel with intestinal inflammation** | **Occurs separately from intestinal inflammation** | **May or may not occur in parallel with intestinal inflammation** |
| --- | --- | --- |
| - Peripheral arthropathy (type 1) - Erythema nodosum - Sweet’s syndrome - Oral aphthous ulcers - Episcleritis | - Axial arthropathy* - Peripheral arthropathy (type 2) | - Pyoderma gangrenosum - Uveitis - Primary sclerosing cholangitis |

*In patients with CD and ankylosing spondylitis, concurrent inflammation in the gut and joints has been reported but the immunological interaction between the two conditions has not been established [244].

## Supplementary Table 5. Crohn’s Disease Activity Index [52]. Modified from Sandborn et al. 2002.

| **Variable** | **Score** | **Multiplier** | **Total** |
| --- | --- | --- | --- |
| No. of liquid or soft stools (each day for 7 days) |  | x 2 |  |
| Abdominal pain, sum of 7 daily ratings | 0 = none  1 = mild  2 = moderate  3 = severe | x 5 |  |
| General well-being, sum of 7 daily ratings | 0 = generally well  1 = slightly under par  2 = poor  3 = very poor  4 = terrible | x 7 |  |
| Number of complications (arthritis or arthralgia, iritis or uveitis, erythema nodosum or pyoderma gangrenosum or aphthous stomatitis, anal fissure or fistula or abscess, other fistula, fever >37.8°C) | 1 for each | x 20 |  |
| Use of diphenoxylate or loperamide for diarrhea | 0 = no  1 = yes | x 30 |  |
| Abdominal mass | 0 = no  2 = questionable  5 = definite | x 10 |  |
| Hematocrit (males: 47-Hct [%], females: 42-Hct [%]) |  | x 6 |  |
| Body weight (1-weight/standard weight) x 100 (add or subtract according to sign) |  | x 1 |  |
| Total CDAI |  |  | (Sum) |
| CDAI, CD activity index; Hct, hematocrit | | | |

## Supplementary Table 6. Harvey-Bradshaw Index [62].

| **Variable** | **Score** | **Total** |
| --- | --- | --- |
| General well-being | 0 = generally well  1 = slightly below par  2 = poor  3 = very poor  4 = terrible |  |
| Abdominal pain | 0 = none  1 = mild  2 = moderate  3 = severe |  |
| No. of liquid stools daily |  |  |
| Abdominal mass | 0 = no  1 = dubious  2 = definite  3 = definite and tender |  |
| Number of complications (arthralgia, uveitis, erythema nodosum, aphthous ulcer, pyoderma gangrenosum, anal fissure, new fistula, abscess) | 1 for each |  |
| Total HBI |  | (Sum) |
| HBI, Harvey-Bradshaw index | | |

## Supplementary Table 7. SAR recommendations for when to use CTE and when to use MRE [83].

| Consider CTE when: | Consider MRE when: |
| --- | --- |
| - There is a concern about sepsis, or a suspicion of complex abdominal penetrating disease with need for potential subsequent intervention - Patient is older (age >35 years) - It is the first cross-sectional exam for acutely symptomatic patients - Needing to rule out other diseases that may cause diarrhea, or to evaluate for other small bowel diseases - Low-dose CT techniques are utilized - The patient has contraindications to MR imaging, allergy to gadolinium-based contrast agents, or claustrophobia during prior MR exams - There is local access and expertise | - The patient has undergone CT previously - The patient is young (<35 years) - Needing to evaluate a patient who is not acutely ill or to assess response to therapy - There is a known perianal fistula or perianal sepsis - The patient is pregnant (perform MRE without intravenous contrast) - There is an allergy to iodinated contrast media - There is local access and expertise |
| CT(E), computed tomography (enterography); MR(E), magnetic resonance (enterography); SAR, Society of Abdominal Radiology. | |

## Supplementary Table 8. Accuracy of cross-sectional imaging techniques for grading CD severity [93].

|  | **CTE** | **MRE** | **US** |
| --- | --- | --- | --- |
| Per-patient accuracy (%) | | | |
| Accurate grading | 86 | 84 | 44 |
| Overgrading | 10 | 9 | 25 |
| Undergrading | 3 | 6 | 31 |
| Per-segment accuracy (%) | | | |
| Accurate grading | 87 | 67-82 | 56-75 |
| CTE, computed tomography enterography; MRE, magnetic resonance enterography; US, ultrasound | | | |

## Supplementary Table 9. Crohn’s disease Endoscopic Index of Severity (CDEIS) [95]. Modified from Mary et al. 1989.

|  | **Ileum** | **Right colon** | **Transverse colon** | **Left colon and sigmoid** | **Rectum** | **Total** |
| --- | --- | --- | --- | --- | --- | --- |
| Deep ulcerations (12 if present, 0 if absent) |  |  |  |  |  | Total 1 |
| Superficial ulcerations (6 if present, 0 if absent) |  |  |  |  |  | Total 2 |
| Surface involved by disease (cm VAS) |  |  |  |  |  | Total 3 |
| Surface involved by ulceration (cm VAS) |  |  |  |  |  | Total 4 |
| Sum of totals 1+2+3+4 | | | | | | Total A |
| Number of segments visualized in part or entirely (from 1 to 5) | | | | | | n |
| Total A/n | | | | | | Total B |
| If ulcerated stenosis in any segment, add 3 | | | | | | Total C |
| If non-ulcerated stenosis in any segment, add 3 | | | | | | Total D |
| Total B + C + D | | | | | | CDEIS score |
| CDEIS, CD endoscopic index of severity; VAS, visual analog scale | | | | | | |

## Supplementary Table 10. Simplified Endoscopic Activity Score for Crohn’s disease (SES-CD) [96]. Modified from Daperno et al. 2004

|  | **Ileum** | **Right colon** | **Transverse colon** | **Left colon and sigma** | **Rectum** | **Total** |
| --- | --- | --- | --- | --- | --- | --- |
| Size of ulcers  0 = none  1 = aphthous (0.1-0.5 cm)  2 = large (0.5-2.0 cm)  3 = very large (>2 cm) |  |  |  |  |  | Total 1 |
| Affected surface  0 = 0%  1 = <50%  2 = 50-75%  3 = >75% |  |  |  |  |  | Total 2 |
| Ulcerated surface  0 = 0%  1 = <10%  2 = 10-30%  3 = >30% |  |  |  |  |  | Total 3 |
| Presence of narrowings  0 = no  1 = single, can be passed  2 = multiple, can be passed  3 = cannot be passed |  |  |  |  |  | Total 4 |
| Sum of totals | | | | | | SES-CD score |

SES-CD, simplified activity score for CD

## Supplementary Table 11. CDEIS and SES-CD scores used to define disease severity [99]. Modified from Kucharski et al. 2016.

|  | CDEIS | SES-CD |
| --- | --- | --- |
| Remission | <3 | 0-2 |
| Mild disease | 3-9 | 3-6 |
| Moderate disease | 10-12 | 7-15 |
| Severe disease | >12 | >15 |

CDEIS, Crohn’s disease endoscopic index of severity; SES-CD, simplified endoscopic activity score for Crohn’s disease

## Supplementary Table 12. Hughes/Cardiff Classification of Anal CD and 1992 Addition [145].

| Ulceration (U) | Fistula/abscess (F) | Stricture (S) |
| --- | --- | --- |
| Not present-0 | Not present-0 | Not present-0 |
| Superficial fissures-1 | Lower/superficial-1 | Reversible stricture (spasm/membranous)-1 |
| a. Posterior and/or anterior | a. Perianal | a. Anal canal-spasm |
| b. Lateral | b. Anovulval, anoscrotal | b. Low rectum-membranous |
| c. With gross skin tags | c. Intersphincteric | c. Spasm with severe pain, no sepsis identified |
|  | d. Anovaginal |  |
| Cavitating ulcers-2 | High/complex-2 | Irreversible stricture (severe fibrotic)-2 |
| a. Anal canal | a. Blind supralevator | a. Anal stenosis |
| b. Lower rectum | b. High direct (anorectal) | b. Extrarectal stricture |
| c. With extension to perineal skin | c. High complex |  |
| (aggressive ulceration) | d. Rectovaginal |  |
|  | e. Ileoperineal |  |
| Addition to Hughes/Cardiff Classification (1992) | | |
| Associated anal conditions (A) | Proximal intestinal disease (P) | Disease activity (in anal locations) (D) |
| None-0 | No proximal disease-0 | Active-1 |
| Hemorrhoids-1 | Contiguous rectal disease-1 | Inactive-2 |
| Malignancy-2 | Colon (rectum spared)-2 | Inconclusive-3 |
| Other (specify)-3 | Small intestine-3 |  |
|  | Investigation incomplete-4 |  |

## Supplementary Table 13. Perianal CD Activity Index (PDAI) [147].

| Item | Score |  |
| --- | --- | --- |
| Discharge | 0 | No discharge |
|  | 1 | Minimal mucous discharge |
|  | 2 | Moderate mucous or purulent discharge |
|  | 3 | Substantial discharge |
|  | 4 | Gross fecal soiling |
| Pain and restriction of activities | 0 | No activity restriction |
|  | 1 | Mild discomfort, no restriction |
|  | 2 | Moderate discomfort, some limitation activities |
|  | 3 | Marked discomfort, marked limitation |
|  | 4 | Severe pain, severe limitation |
| Restriction of sexual activity | 0 | No restriction sexual activity |
|  | 1 | Slight restriction sexual activity |
|  | 2 | Moderate limitation sexual activity |
|  | 3 | Marked limitation sexual activity |
|  | 4 | Unable to engage in sexual activity |
| Type of perianal disease | 0 | No perianal disease or skin tags |
|  | 1 | Anal fissure of mucosal tear |
|  | 2 | <3 Perianal fistulas |
|  | 3 | >3 Perianal fistulas |
|  | 4 | Anal sphincter ulceration of fistula with significant undermining of skin |
| Degree of induration | 0 | No induration |
|  | 1 | Minimal induration |
|  | 2 | Moderate induration |
|  | 3 | Substantial induration |
|  | 4 | Gross fluctuance or abscess |
| Total |  |  |
| PDAI, perianal CD activity index. | | |

## Supplementary Table 14. Reported sensitivity and specificity of different cross-sectional imaging techniques for the diagnosis of stricturing CD [175, 179].

|  | **Sensitivity (%)** | **Specificity (%)** |
| --- | --- | --- |
| Ultrasound |  |  |
| Transabdominal ultrasound | 73-100 | 63-100 |
| Small intestinal contrast ultrasound (SICUS) | 92-97.5 | 0-100 |
| SICUS with power Doppler | 88 | 88 |
| Computed tomography (CT) |  |  |
| CT enteroclysis | 92.3 | 38.9 |
| CT enterography | 85-100 | 100 |
| Hybrid PET/CT enterography | 85 | NR |
| Magnetic resonance (MR) imaging |  |  |
| MR enterography | 75-100 | 91-100 |
| High resolution MR enterography | 86 | 95 |
| Hybrid PET/MR enterography | 85 | NR |

CT, computed tomography; MR, magnetic resonance; NR, not reported; PET, positron emission tomography; SICUS, small intestinal contrast ultrasound

## Supplementary Table 15. Definitions for patients at high risk of recurrence after resection

| **POCER study [185].** | **UK guidelines [1].** | **French guidelines [6].** | **Review by Lightner & Shen [202].** |
| --- | --- | --- | --- |
| Any: | Two or more: | Two or more^a^: | Two or more^b^: |
| - Current smoking | - Current smoking | - Current smoking | - Current smoking |
| - Penetrating disease | - Penetrating disease | - Penetrating disease | - Penetrating disease |
| - Previous resection | - Previous resection | - Previous resection | - Previous resection |
|  | - Perianal fistula | - Fistulizing phenotype (Montreal B3 [penetrating phenotype]) | - Perianal disease at diagnosis |
|  | - Extensive small bowel disease | - Length of intestinal resection >50 cm | - Age <30 years at diagnosis |
|  | - Residual active disease |  | - Time since previous surgery <3 years |
|  | - Granulomas or myenteric plexitis |  |  |

^a^One risk factor indicates moderate risk

^b^One or two risk factors indicates moderate risk.

## Supplementary Table 16. Modified Rutgeerts score [213].

| **Score** | **Endoscopic findings** |
| --- | --- |
| i0 | Absence of any lesions at the site of the anastomosis and in the neoterminal ileum |
| i1 | ≤5 aphthous ulcers |
| i2 | >5 aphthous ulcers with normal mucosa between the lesions, or skip lesions, or lesions confined to the ileocolonic anastomosis |
| i2a | Lesions confined to the ileocolonic anastomosis, including anastomotic stenosis |
| i2b | >5 aphthous ulcers or larger lesions with intact mucosa in between, as well as for lesions found in the neo-terminal ileum, with or without anastomotic lesions |
| i3 | Diffuse aphthous ileitis with diffusely inflamed mucosa |
| i4 | Diffuse inflammation with large ulcers (≥5 mm), nodules and/or narrowing |

## Supplementary Table 17. Relapse rates associated with discontinuation of anti-TNFα therapy in clinical studies of CD

| **Author, year** | **Study type** | **Patients (therapy)** | **Probability/rate of relapse** | **Effect of concomitant IM therapy** |
| --- | --- | --- | --- | --- |
| Louis, 2012 [225]. | Prospective observational | N=115 (IFX); n=19 (MTX); n=96  (AZA/6-MP) | 12 months: 44%; 24 months: 52% | Not reported |
| Kennedy, 2016 [227]. | Retrospective observational | N=146 (ADA/IFX); n=66 (AZA); n=9 (6-MP); n=20 (MTX) | 12 months: 36%; 24 months: 56% | IM at withdrawal led to non-significant decrease in relapse risk (HR=0.68; p=0.101) |
| Casanova, 2017 [224]. | Retrospective observational | N=731 (ADA/IFX); n=232 (thiopurine); n=6 (MTX) | 12 months: 22% | IM at withdrawal significantly decreased relapse risk (HR=0.67; p=0.003) |
| Casanova, 2021 [230]. | Retrospective, observational | N=1055 | 12 months: 19%; 24 months: 31%; 36 months: 38%; 48 months: 44%; 60 months: 48%; | Remission was regained in a high proportion of patients following retreatment with other therapies, including IM |
| Molander, 2014 [228]. | Prospective observational | N=17 (ADA/IFX); n=12 (AZA/6-MP) | 12 months: 29% | No apparent effect |
| Song, 2021 [231] | Retrospective, observational | N=71 (ADA/IFX) | 12 months: 11%; 24 months: 31%; 36 months: 47%; 48 months: 63%, | IM at withdrawal led to non-significant decrease in relapse risk (HR=0.70; p=0.411) |

6-MP, 6-mercaptopurine; ADA, adalimumab; AZA, azathioprine; HR, hazard ratio; IFX, infliximab; IM, immunomodulator; MTX, methotrexate.

## Supplementary Table 18. Relapse rates associated with discontinuation of azathioprine monotherapy in clinical studies of CD

| **First author, year** | **Study type** | **Patients** | **Probability/rate of relapse** | **Risk factors associated with relapse** |
| --- | --- | --- | --- | --- |
| O’Donoghue, 1978 [234]. | RCT | N=51 (AZA, placebo) | Discontinued AZA: 6 months, 25%; 12 months, 41% Continued AZA: 6 months, 0%; 12 months, 5% | Study failed to identify any subgroup or factors associated with greater or lesser response |
| Vilien, 2004 [236]. | RCT, OL | N=29 (AZA, no AZA) | Discontinued AZA: 12 months, 53% Continued AZA: 12 months, 15% | Higher AZA dose associated with increased relapse rate |
| Lemann, 2005 [233]. | RCT | N=83 (AZA, placebo) | Discontinued AZA: 18 months, 21% Continued AZA: 18 months, 8% | CRP ≥20 mg/L, hemoglobin <12 g/dL, time without steroids <50 months were independent factors associated with an increased relapse rate |
| Wenzl, 2015 [237]. | RCT | N=52 (AZA, placebo) | Discontinued AZA: 24 months, 31% Continued AZA: 24 months, 15% | Higher AZA dose associated with increased relapse rate |
| Bouhnik, 1996 [232]. | Retrospective, OL | N=157 (AZA/6MP vs no AZA/6MP) | Discontinued AZA/6MP: 12 months, 38%; 60 months, 75% Continued AZA/6MP: 12 months, 11%; 60 months, 32% | Younger age (<26 years), male gender, withdrawal <4 years after remission associated with increased relapse rate |
| Treton, 2009 [238]. | OL extension of Lemann, 2005 | N=66 (no AZA) | Discontinued AZA: 12 months, 14%; 36 months, 53%; 60 months, 63% | CRP ≥20 mg/L, neutrophil count ≥4.0 x 10^9^/L, and hemoglobin <12 g/dL independently associated with an increased relapse rate |
| Sokol, 2010 [235]. | Retrospective review | N=141 (AZA [controls] vs no AZA) | Discontinued AZA: 24 months, 57%; 60 months, 73% Continued AZA: 24 months, 18%; 60 months, 44% | Male gender, absence of smoking associated with an increased relapse rate |

6MP, 6-mercaptopurine; AZA, azathioprine; CRP, C-reactive protein; OL, open-label; RCT, randomized controlled trial.

## Supplementary Table 19. Relapse findings associated with discontinuation of an immunomodulator from combination therapy with an anti-TNF α agent in clinical studies of CD

| **First author, year** | **Study type** | **Patients** | **Relapse findings** |
| --- | --- | --- | --- |
| Van Assche, 2008 [243]. | RCT, OL | N=80 (continued / discontinued IM) | Similar proportion of patients who continued or discontinued immunomodulator at 6 months needed a change in infliximab dosing interval or stopped infliximab therapy at 2 years |
| Oussalah, 2010 [241]. | Observational | N=48 | AZA discontinuation associated with a high risk of relapse in patients with a duration of combination therapy of <27 months |
| Roblin, 2017 [242]. | RCT, OL | N=81 (continued, reduced, discontinued AZA) | Clinical relapse or change in therapy was higher in patients who discontinued AZA (30.8%) compared with those who continued (17.9%) or reduced the dose of AZA (11.1%). Dose reduction appears as effective as continuation in patients receiving combination therapy. |
| Hisamatsu, 2019 [240]. | RCT, OL | N=50 (continued / discontinued AZA) | Continuation of AZA >6 months offers no clear benefit over scheduled anti-TNFα monotherapy in terms of clinical remission |

AZA, azathioprine; IM, immunomodulator; OL, open-label; RCT, randomized controlled trial; TNF, tumor necrosis factor.

## Supplementary Figure 1. Schema of simple and complex fistula anatomy (modified from Parks classification for consistency with AGA classification) [144].


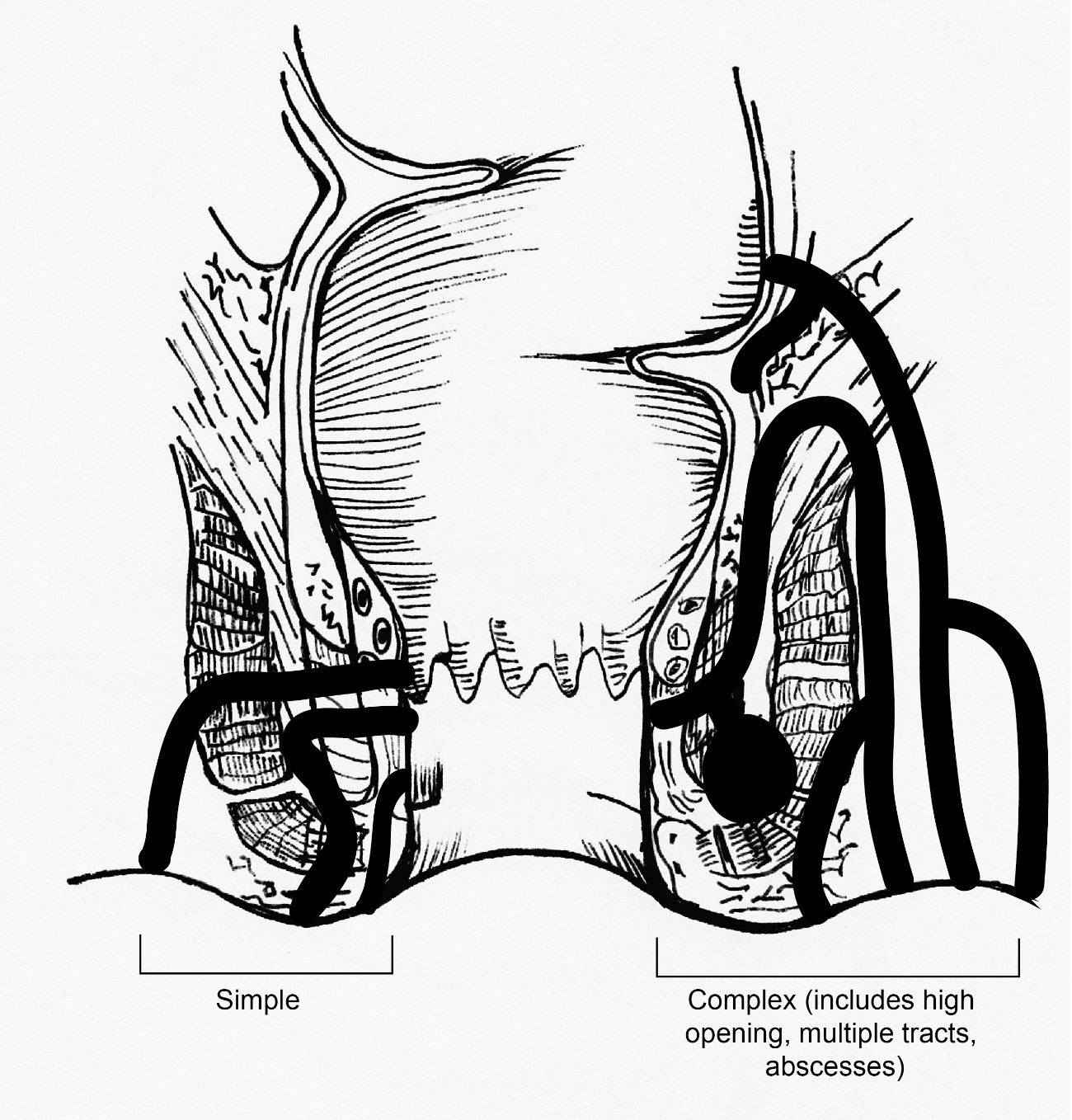

Supplement: Supplementary file 1 — Supplementary file1 (DOCX 622 KB) [file 535_2023_1958_MOESM1_ESM.docx]
